# Metalloprotease Gp63-Targeting Novel Glycoside Exhibits Potential Antileishmanial Activity

**DOI:** 10.3389/fcimb.2022.803048

**Published:** 2022-05-04

**Authors:** Amrita Chakrabarti, Chintam Narayana, Nishant Joshi, Swati Garg, Lalit C. Garg, Anand Ranganathan, Ram Sagar, Soumya Pati, Shailja Singh

**Affiliations:** ^1^ Department of Life Sciences, School of Natural Sciences, Shiv Nadar University, Greater Noida, India; ^2^ Department of Chemistry, School of Natural Sciences, Shiv Nadar University, Greater Noida, India; ^3^ Special Centre for Molecular Medicine, Jawaharlal Nehru University (JNU), New Delhi, India; ^4^ Gene Regulation Laboratory, National Institute of Immunology, New Delhi, India; ^5^ Department of Chemistry, Institute of Science, Banaras Hindu University, Varanasi, India

**Keywords:** *Leishmania*, Glycoside 2, LdGp63, post kala-azar dermal leishmaniasis (PKDL), cellular thermal shift assay (CETSA)

## Abstract

Visceral leishmaniasis (VL) and post kala-azar dermal leishmaniasis (PKDL) affect most of the poor populations worldwide. The current treatment modalities include liposomal formulation or deoxycholate salt of amphotericin B, which has been associated with various complications and severe side effects. Encouraged from the recent marked antimalarial effects from plant-derived glycosides, in this study, we have exploited a green chemistry-based approach to chemically synthesize a library of diverse glycoside derivatives (Gly1–12) and evaluated their inhibitory efficacy against the AG83 strain of *Leishmania donovani*. Among the synthesized glycosides, the *in vitro* inhibitory activity of Glycoside-2 (Gly2) (1.13 µM IC50 value) on *L. donovani* promastigote demonstrated maximum cytotoxicity with ~94% promastigote death as compared to amphotericin B that was taken as a positive control. The antiproliferative effect of Gly2 on promastigote encouraged us to analyze the structure–activity relationship of Gly2 with Gp63, a zinc metalloprotease that majorly localizes at the surface of the promastigote and has a role in its development and multiplication. The result demonstrated the exceptional binding affinity of Gly2 toward the catalytic domain of Gp63. These data were thereafter validated through cellular thermal shift assay in a physiologically relevant cellular environment. Mechanistically, reduced multiplication of promastigotes on treatment with Gly2 induces the destabilization of redox homeostasis in promastigotes by enhancing reactive oxygen species (ROS), coupled with depolarization of the mitochondrial membrane. Additionally, Gly2 displayed strong lethal effects on infectivity and multiplication of amastigote inside the macrophage in the amastigote–macrophage infection model *in vitro* as compared to amphotericin B treatment. Gp63 is also known to bestow protection against complement-mediated lysis of parasites. Interestingly, Gly2 treatment enhances the complement-mediated lysis of *L. donovani* promastigotes in serum physiological conditions. In addition, Gly2 was found to be equally effective against the clinical promastigote forms of PKDL strain (IC50 value of 1.97 µM); hence, it could target both VL and PKDL simultaneously. Taken together, this study reports the serendipitous discovery of Gly2 with potent antileishmanial activity and proves to be a novel chemotherapeutic prototype against VL and PKDL.

**Graphical Abstract:**
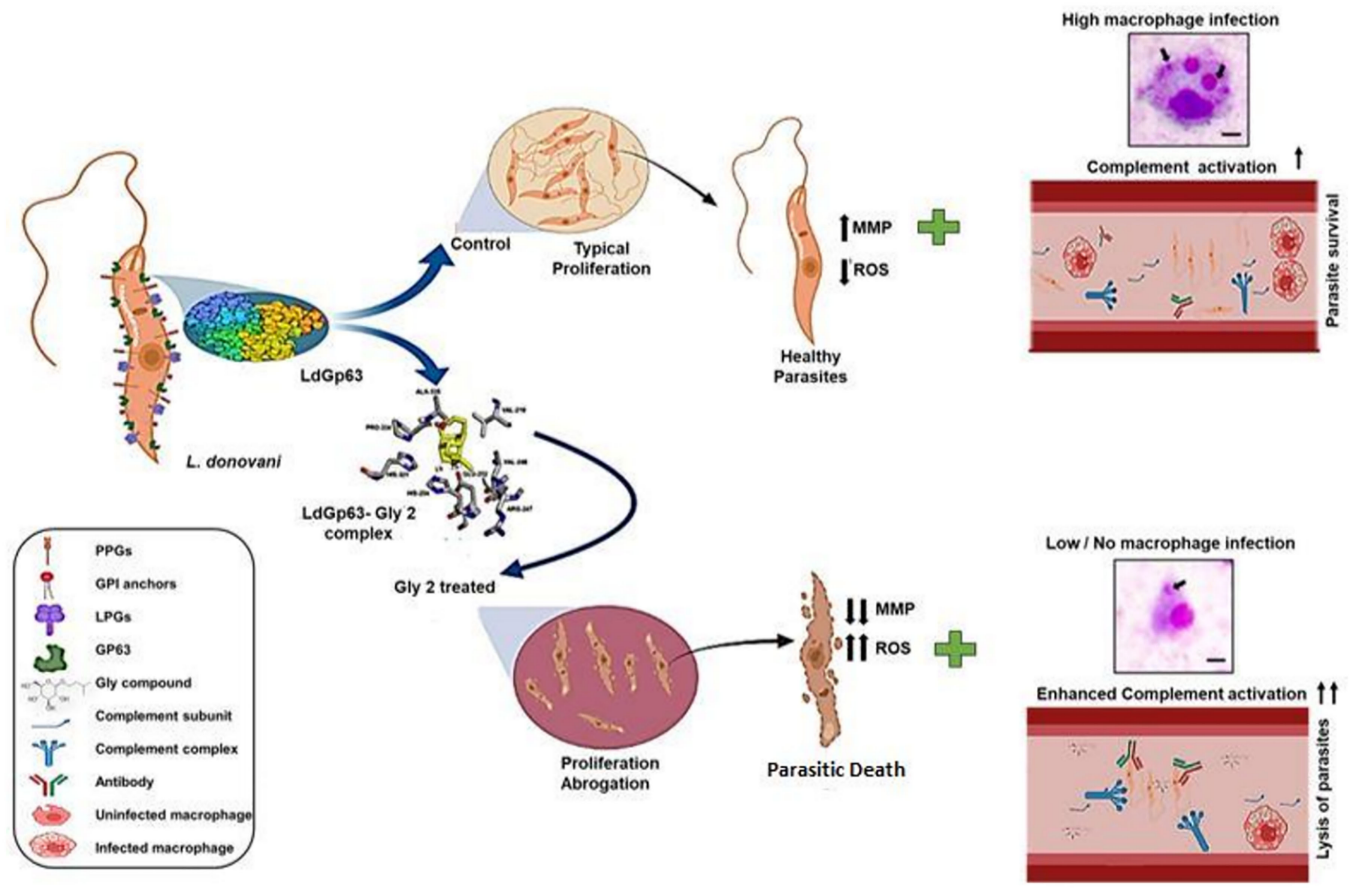


## Introduction

Leishmaniasis is a globally neglected vector-borne disease transmitted by the bite of a female sand fly infected with the protozoan parasite of the genus *Leishmania*. It has many clinical manifestations ranging from a mild cutaneous variant to the life-threatening visceral form (Arenas et al., 2017). Patients cured of visceral leishmaniasis (VL) are prone to post kala-azar dermal leishmaniasis (PKDL), an aggressive clinical manifestation of VL (Zijlstra, 2016). Leishmaniasis, caused by *L. donovani*, is endemic in several parts of the tropics, subtropics, and southern Europe. The disease is a severe threat to 350 million people, with a prevalence of 12 million people worldwide (Oryan and Akbari, 2016). Nearly 0.7–1 million newly reported cases of leishmaniasis span about 100 endemic countries (Burza et al., 2018). An estimated 50,000–90,000 new cases of VL arise annually, majorly in parts of Brazil, East Africa, and India, only 25%–45% of which get reported to WHO (Bi et al., 2018). In Brazil, the average annual age-adjusted mortality rate stands at 0.15 deaths per 100,000 individuals, and the case fatality rate is 8.1% (Martins-Melo et al., 2014). Thus, to target this fatal disease, WHO has already declared the development of new treatments of utmost priority.

Over several years, the available diagnosis and treatment modalities for VL relied on pentavalent antimonials, but unfortunately, these drugs are found to be unsatisfactory in combating the disease, as evidenced by increasing disease burden due to high toxicity, delayed drug release and response, and inimical side effects (Croft et al., 2006; Ponte-Sucre et al., 2017). In the conquest of identifying better leishmanicidal compounds, plant-derived and mimicking products are gaining the attention of researchers. Products from aloe vera, luteolin, quassin, berberine chloride, and artemisinin are very prominent. These derivatives have a similar mode of action and induce oxidative stress leading to parasite death. Despite being plant derivatives, these drugs also depicted low efficacy with certain side effects (Sen and Chatterjee, 2011; Singh et al., 2016). This limited efficacy of currently available drugs demands efficient drug development strategies involving both target identification and validation in the pathogenesis of VL. Previous studies elucidated the role of various *Leishmania* spp. proteases involved in parasite life cycle and pathogenesis. Notably, leishmanolysin of *Leishmania* spp., the known zinc metalloprotease, Gp63 has been identified as an important multifunctional virulence factor, found in abundance on the surface of promastigotes and in limited quantities inside the parasite (Etges et al., 1985; Yao et al., 2003; Sunter and Gull, 2017). It is the key enzyme responsible for parasite propagation, promastigote binding and internalization in macrophages, and attenuation of reactive oxygen intermediate formation that favors amastigote proliferation (Kamhawi, 2006). The myriad diversity as well as the high catalytic activity at the body’s physiological temperature of this virulence factor favors the dissemination of the parasite in the host (Chaudhuri and Chang, 1988; Yao et al., 2002; McGwire et al., 2003; Yao et al., 2003). Recent reports from certain parasitic models demonstrated the protective nature of Gp63 against complement fixation and processing that shields *Leishmania* promastigotes during its tarriance into mammalian hosts (Brittingham et al., 1995; Joshi et al., 1998; Joshi et al., 2002). Emerging studies have shown that plant-derived glycoside formulations from Asteraceae extracts can inhibit protozoan parasites such as *Leishmania*, *Plasmodium*, *Trypanosoma*, and intestinal worms. Recently, oral administration of plant-derived beta-glycosides, such as esculin and amygdalin, to sand fly *Lutzomyia longipalpis*, the main vector of American VL, has shown to drastically affect the sand fly physiology and *Leishmania* development, thus proposed as promising transmission-blocking sugar baits. Additional study has identified promising antibacterial and antiparasitic activity of oleanolic acid and its glycosides isolated from marigold (*Calendula officinalis*) (Szakiel et al., 2008).

Based on this evidence, we strategized to develop a purely non-toxic and non-hazardous form of glycoside with no effect on human health or environment. Considering the green chemistry approach, we have designed and synthesized a library of novel glycoside derivatives with D-glucose and N-acetyl-D-glucosamine as the primary backbone template. Among these, **Gly2** showed promising results against both *L. donovani* promastigote and intra-macrophagic amastigote forms. **Gly2** treatment led to abrogation of parasite multiplication, induction of reactive oxygen species (ROS) generation, and disruption of mitochondrial membrane potential leading to promastigote death, suggesting its therapeutic implication against VL. Besides its inhibitory role in cultured parasites *in vitro*, **Gly2** demonstrated efficacy in the prevention of parasite evasion from complement-mediated lysis in an *in vitro* model mimicking the body’s physiological condition. The structure–activity relationship (SAR) analysis of novel glycosides, along with cellular thermal shift assay (CETSA) and *in silico* studies, revealed efficient binding of **Gly2** molecule with Gp63 catalytic domain. Additionally, **Gly2** demonstrated excellent antileishmanial activity against the clinical isolate of PKDL. Overall, our work has discovered a purely non-toxic glycoside derivative with strong antileishmanial potential.

## Materials and Methods

### Parasite Growth and Maintenance

Promastigote forms of *L. donovani* (MHOM/IN/1983/AG83) were cultured at 26°C in M199 (GIBCO, India), pH 7.4, supplemented with 10% (v/v) inactivated fetal bovine serum (FBS, GIBCO, India) and 0.02 mg/ml gentamycin (Life Technologies, USA). The clinical isolate of PKDL, BS12, was obtained as a gift from Prof. Mitali Chatterjee [Department of Pharmacology, Institute of Postgraduate Medical Education and Research (IPGMER), Kolkata, India]. BS12 was routinely cultured at 22°C in M199 (GIBCO, India) with 100 U/ml penicillin-streptomycin (Gibco, Invitrogen, Thermo Fisher Scientific, NY, USA), 8 μM hemin (4 mM stock made in 50% triethanolamine) (Sigma, USA), 25 mM 4-(2-hydroxyethyl)-1-piperazineethanesulfonic acid (HEPES), supplemented with 10% heat-inactivated FBS (GIBCO, India). Cultures were maintained between 10^6^ and 10^7^ cells/ml for continuous exponential growth. In addition, 1 × 10^6^ cells/ml parasite count was constantly maintained for all the experiments (Ramu et al., 2017).

### Cell Culture

The J774.A1 murine macrophage cells were grown in Roswell Park Memorial Institute (RPMI) 1640 medium in the presence of 10% (v/v) FBS and 100 U/ml penicillin−100 µg/ml streptomycin (Gibco, Invitrogen, Thermo Fisher Scientific, NY, USA) at 37°C (humidified) and 5% CO_2_. Primary Madin–Darby canine kidney (MDCK) cells were obtained from the National Centre for Cell Science, Pune, India. MDCK cells were derived from the kidney tissue of an adult female Cocker Spaniel. These were grown in Dulbecco’s modified Eagle’s minimal essential medium (DMEM) in the presence of 10% (v/v) FBS and 100 U/ml penicillin−100 µg/ml streptomycin (Gibco, Invitrogen, Thermo Fisher Scientific, NY, USA) at 37°C (humidified) and 5% CO_2_ (Ayana et al., 2018; Shivappagowdar et al., 2021).

### Synthesis of Glycoside Derivatives

Pre-stirred solution of glucose (200 mg, 1.11 mmol) or glucosamine (200 mg, 1.11 mmol) was prepared in neat alcohol (5–10 ml), and Amberlite IR 120-H^+^ resin (400 mg) was added to it. The resulting mixture was stirred at 100°C for 24 h. After completion of the reaction, the mixture was cooled down to room temperature and filtered to remove the resin. The filtrate was evaporated under reduced pressure to obtain compounds 1-3 as a white solid in acceptable to good yield. Compounds 4–6 were prepared in good yield adopting a similar reaction protocol using the corresponding alcohol and glucose or glucosamine followed by acetylation using acetic anhydride in pyridine at room temperature. All the final compounds 1–6 were purified using flash column chromatography before using them for biological activity. The final compounds were formed as semisolid or solid and were characterized by ^1^H-NMR and ^13^C-NMR. ^1^H-NMR and ^13^C-NMR spectra in CD_3_OD and D_2_O were recorded on Bruker 400 MHz spectrometer at ambient temperature. Here, ^1^H was recorded by 400 MHz and ^13^C was recorded by 100 MHz. Proton chemical shifts are given in ppm relative to the internal standard (tetramethylsilane) or referenced relative to the solvent residual peaks (CD3OD: δ 3.31). Multiplicity was denoted as follows: s (singlet), d (doublet), t (triplet), q (quartet), m (multiplet), dd (doublet of doublet), dt (doublet of triplet), td (triplet of doublet), ddd (doublet of doublet of doublet), etc. Coupling constants (J) were reported in Hz. Column chromatography was performed by using silica gel 100–200 and 230–400 mesh. High Resolution Mass Spectrometry (HRMS) spectra were determined from quadrupole/Q-TOF mass spectrometer with an ESI source (**Supplementary Material**) (Yu et al., 2004).

### Treatment Procedure With Synthesized Glycosides

All the compounds were dissolved in dimethyl sulfoxide (DMSO) (Sigma-Aldrich) for 1 mM stock solution and were screened against promastigotes at a concentration of 5 µM. For Glycosides (Gly) 2, 6, and 8, a range of concentrations starting from 100 nM to 100 µM were screened against promastigote forms of parasite and mammalian cells. Parasites (AG83 and PKDL strain) treated with amphotericin B (3 µg/ml) (Sigma-Aldrich) were maintained as the positive control.

### Anti-Parasite Drug Susceptibility Assay

AG83 strain of *L. donovani* were exposed to various Gly compound concentrations (100 nM–100 µM) in a 96-well microtiter plate (100 µl/well volume) and were incubated for 72 h at 26°C. Finally, lactate dehydrogenase (LDH) cytotoxic assay was performed as per standard protocol (CytoTox 96 Non-Radioactive Cytotoxicity Assay-Promega, USA). The percent cytotoxicity of test compounds was calculated by normalizing with amphotericin B as 100%. Further percentage growth inhibition was calculated using the formula:

% Growth Inhibition = Gly treated LDH activity- Spontaneous LDH activity

/Maximum LDH activity- Spontaneous LDH activity * 100

As per the formula, the spontaneous LDH Activity = activity of the untreated cells and the maximum LDH activity = activity of the amphotericin B-treated cells. IC50 value for Gly2, 6, and 8 treated was generated for AG83 and PKDL strain of *L. donovani* using sigmoidal dose–response model with nonlinear regression tool (Ramu et al., 2017).

### Qualitative Estimation of Live/Dead Parasites

The toxic effect of the three compounds, Gly2, 6, and 8 on promastigotes at 5 µM >IC50 concentration was measured by Hoechst and propidium iodide (PI) double staining. After treatment with the compounds for 24 h, parasites were harvested, washed in 0.01 M PBS (pH = 7.4), and stained with Hoechst 33258 (1 µg/ml) (Life Technologies, USA) and PI (5 µg/ml) (Life Technologies, USA) followed by incubation for a period of 20 min at 37°C. Subsequently, cells were washed for excessive stain removal and visualized using fluorescence microscope with 510–560-nm filter block for PI red fluorescence and excitation/emission spectra at 361/497 for Hoechst (López-Arencibia et al., 2015).

### *In Silico* Studies

The physicochemical properties of compounds were calculated using SwissADME (Daina et al., 2017) module provided in SIB (Swiss Institute of Bioinformatics) web server (https://www.sib.swiss). The designed **Gly** compounds were evaluated for their ADME profile, including drug-likeness, partition coefficient, solubility, and oral bioavailability parameters according to Lipinski’s “rule-of-five” (Daina et al., 2017).

### Antibody Generation

Synthetic oligonucleotides encoding the catalytic motif of LdGp63 with PstI and HindIII overhangs were synthesized and ligated to PstI and HindIII-digested pQELTB plasmid and transformed into *Escherichia coli* DH5α cells for propagation and into *E. coli* M15 cells for recombinant fusion protein expression. *E. coli* M15 cells containing the recombinant plasmid were induced with 1 mM Isopropyl β-D-1-thiogalactopyranoside (IPTG). The recombinant fusion protein was purified to near homogeneity from solubilized inclusion bodies using metal affinity chromatography, taking advantage of the histidine tag present at the N-terminal of the fusion protein. Female BALB/c mice were immunized with the purified fusion protein followed by a single booster on day 15 post immunization. Blood was obtained a week post booster administration through the retro-orbital plexus. Anti-fusion protein antiserum was obtained after incubating the blood at Room Temperature (RT) for 1 h followed by centrifugation at (1,957 g, 10 min) at 4°C. Antiserum thus prepared was stored at -80°C until further use (Nejad-Moghaddam and Abolhassani, 2009).

### Western Blot Analysis

Immunoblotting assay was performed using generated mouse polyclonal anti-LdGp63 catalytic domain, which were used at 1:500 dilution. The whole-parasite lysates were washed twice with Phosphate Buffer Saline (PBS), lysed in lysis buffer (50 mM HEPES, 150 mM NaCl, 1% Triton X-100 and 1% IGEPAL, 1 mM phenylmethyl sulfonyl fluoride), and denatured at 70°C. The samples were then homogenized with a 1-ml syringe and a 22G needle before Sodium dodecyl-sulfate polyacrylamide gel electrophoresis (SDA-PAGE) and blotting onto nitrocellulose membranes with 0.2-µm pore size. The membranes were blocked with 5% skimmed milk powder dissolved in PBS containing 0.1% Tween-20. After incubation for 2 h at room temperature, the blot was subsequently washed with PBS and later was probed with primary generated mouse polyclonal anti-LdGp63 antibody and rabbit IgG conjugated to horseradish peroxidase as secondary antibody. After incubation, the membrane was washed thrice with PBS, and the signals were observed under Enhanced Chemiluminescence (ECL)-exposed X-ray film at room temperature in a dark room having safe red light (Jost et al., 2011).

### Confocal Imaging of Gp63 Localization in Promastigotes

Immunofluorescence assay was performed with anti-LdGp63 catalytic domain antibody that was used at 1:500 dilution. The smears of the parasites were stained with indicated primary antibodies and using secondary antibodies labeled with Alexa Fluor 488 (Invitrogen). Coverslips were mounted with ProLong Gold Anti fade DAPI (Invitrogen), and images were collected with an Olympus 1000 microscope (Cuevas et al., 2003).

### Promastigote Proliferation Assay

AG83 parasite growth and multiplication were assessed by fluorescence-activated cell sorting and fluorescence microscopy with 6-carboxyfluorescein diacetate succinimidyl ester (CFDA-SE, Life Technologies, USA) as a probe. Promastigotes were washed thrice with PBS. The cells were labeled with CFDA-SE dye and were then incubated at 37°C for 10 min. These cells were then resuspended in ice-cold M199 medium. Furthermore, they were centrifuged at 1,200 g for 10 min (4°C) and resuspended in fresh medium. Cells were treated with the **Gly2** and were analyzed through BD FACS DIVA for 3 consecutive replicates after 0, 24, 48, and 72 h, respectively (Messaritakis et al., 2010).

### Estimation of Reactive Oxygen Species Levels

The redox homeostasis of promastigotes was monitored both qualitatively and quantitively by 2′,7′-dichlorodihydrofluorescein diacetate (DCFDA) (Life Technologies, USA) staining. The untreated and **Gly2**-treated promastigotes (1 × 10^6^ cells/ml) were cultured for 72 h. Following incubation, the parasites were harvested, washed with PBS, and stained with H_2_DCFDA (20 µM) for 20 min at 37°C. Excess stain was removed by washing with PBS, and samples were resuspended in PBS (50 µl) followed by imaging. Fluorescence intensity was determined using an excitation filter at 485 nm and an emission filter at 535 nm using confocal microscope (Nikon Ti eclipse, USA). The samples were also analyzed through BD FACS diva (Shivappagowdar et al., 2019).

### Quantification of Mitochondrial Membrane Potential

Mitochondrial membrane (Δψm) potential was examined using 5,6-dichloro-2-[3-(5,6-dichloro-1,3-diethyl-1,3-dihydro-2H-benzimidazol-2-ylidene)-1propenyl]-1,3-diethyl-,iodide (JC-1 dye) (Life Technologies, USA) as a probe. Treated and untreated groups were incubated for 24 h. Cells were washed with PBS and JC-1 labeled, and samples were analyzed through FACS diva (Beckton-Dickinson, Pharmingen). The approximate fluorescence excitation/emission maxima of 514/529 nm for monomeric form and 585/590 nm for J-aggregate form were used. The labeled cells were also allowed to adhere to glass slides for visualization under fluorescence microscope (Nikon Ti eclipse, USA) (Shivappagowdar et al., 2019).

### Detection of Complement-Mediated Cytolysis of Promastigotes by Uptake of Propidium Iodide

Parasites with an optimum 10% non-heat-inactivated normal human serum (NHS) were incubated with **Gly2** at 5-µM concentration for 15 min. Promastigote lysis was detected by uptake of PI, by use of a FACS Diva flow cytometer (Beckton-Dickinson, Pharmingen), in accordance with the manufacturer’s protocol. Promastigotes were washed for excessive stain removal and filter block for PI red fluorescence, 510–560 nm was used (Moreno et al., 2010).

### *In Silico* Membrane Binding Studies and Docking Studies

Using PerMM web server for theoretical assessment of passive permeability of molecules across the lipid bilayer, we checked the membrane binding studies with synthesized glucoside compounds (Lomize et al., 2019). The three-dimensional (3D) protein structure of *L. major* GP63 (PDB ID- 1lml) was taken as template, and BLASTp with LdGp63 was performed to achieve sequence identity, followed by homology modeling. PROCHECK server was utilized to obtain Ramachandran plot (Laskowski et al., 1993). The chemical structures of compounds were synthesized through the ChemSketch (Spessard, 1998). Protein and ligand structures were optimized using Swiss PDBviewer and ChemBioDraw ultra version 12.0, respectively (Kaplan and Littlejohn, 2001; Cousins, 2011). Autodock version 4.2 and Cygwin terminal were utilized to execute the docking commands (Racine, 2000; Morris and Lim-Wilby, 2008). Binding site for the ligand was chosen around its catalytic domain residues. Chimera version 1.13.1, Ligplot version 2.2, Discovery Studio version 19.1.0, and Pymol version 2.3.2 (De Lano, 2002) software were used for further analysis of docking results [Accelrys Software Inc. (2009), Discovery Studio Modeling Environment, Release 2.5.1, San Diego, CA; crystallography and 2002; Pettersen et al., 2004; Laskowski and Swindells, 2011; Studio, 2015].

### Cellular Thermal Shift Assay

For the CETSA in promastigotes, parasites were seeded in 6-well cell culture plates (1.0 × 10^6^ cells/well) and exposed to Gly2 at the 5 µM >IC50 for 24 h in the incubator. Control cells were incubated with an equal volume of a vehicle. Following incubation, the parasites were washed with **PBS** to remove excess drug and harvested in 200 μl of a lysis solution (50 mM HEPES, 150 mM NaCl, 1% Triton X-100 and 1% IGEPAL, 1 mM phenylmethyl sulfonyl fluoride). The lysates were centrifuged at 14,000 g for 20 min at 4°C, and supernatants were transferred to new tubes. Furthermore, the protein concentration was measured using the Pierce BCA protein assay kit. After preparation of lysates, 30 μl (0.8 mg/ml) aliquots of the supernatants were heated individually on a Thermomixer compact (Eppendorf) at different temperatures for 7 min and then cooled at room temperature for 3 min. CETSA samples were separated by SDS-PAGE, and immunoblotting was performed as described previously using a mouse polyclonal anti-LdGp63 catalytic domain antibody (1:500) (Chakrabarti et al., 2021).

### Inhibitory Activity of Compounds Against Intracellular Amastigotes

For evaluating the efficacy of compounds against intramacrophage amastigotes, Giemsa staining (Sigma-Aldrich) was performed. J774.A1 murine macrophage cells were plated at a cell density of 5 × 10^5^ cell/well in a 6-well flat bottom plate. The cells were infected with late-stage *L. donovani*, rich in promastigotes at a ratio of 10:1. After 6 h, uninfected promastigotes were washed off with PBS. Infected macrophages were treated with compounds at their respective IC50 concentrations and incubated for 24 h for visualization and counting of intracellular parasite load (Ayana et al., 2018).

### Ethics Statement

NHS was obtained from Rotary Blood Bank, Tughlakabad, New Delhi. All methods were carried out in accordance with relevant guidelines and regulations. Ethics committees of Shiv Nadar University approved all of the experiments carried out with NHS. The Animal Ethics Committee (IAEC Code #288/11) of the Jawaharlal Nehru University approved animal usage and procedures for the generation of antibody. The clinical isolate of PKDL, BS12, has been obtained as a kind gift from Prof. Mitali Chatterjee’s Laboratory [Department of Pharmacology, Institute of Postgraduate Medical Education and Research (IPGMER), Kolkata, West Bengal, India]. This strain was cultured in BSL2 lab facility under guidelines of the institutional biosafety committee, Shiv Nadar University.

### Statistical Analysis

Student’s t-test was performed to evaluate significant differences between treatment and control samples in all of the experiments performed using ANOVA test. P-values <0.05 and <0.01 were considered to be significant, indicated as * and **, respectively. Results represent the mean ± SD of minimum three independent experiments. Calculated IC50 value and all statistical analyses were performed using GraphPad Prism version 8.01. Intensity profiles for Western blots were calculated using ImageJ software (Schneider et al., 2012).

## Results

### Synthesis of Glycoside-Based Compounds Using a Green Synthetic Route

**Figure 1:**
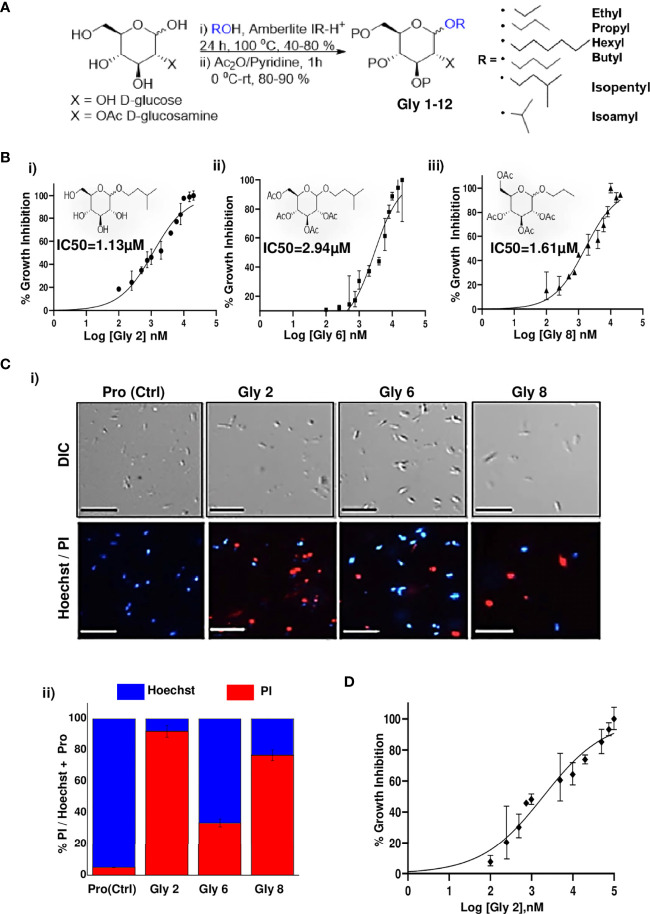
Synthesis scheme of glycoside derivatives and concentration-dependent effects of potent novel glycosides on promastigotes of *Leishmania donovani* AG83 strain with variable membrane binding affinities. **(A)** Synthesis of designed glycosides was carried out with commercially available D-glucose andD-glucosamine coupling with various short- to long-chain alcohols under acidic conditions. **(B)** Percentage inhibition of promastigotes treated with Gly2, 6, and 8 was evaluated using LDH assay for 72 h and plotted as sigmoidal curve. Data normalization was done by taking into consideration the cytotoxicity induced by positive control (amphotericin B 3 µg/ml) as 100%. IC50 values for promastigotes of the AG83 strain were analyzed using GraphPad Prism, represented as the mean ± SD where n = 3, independent experiments. **(C)** (i) The representative screenshots of propidium iodide (PI)- and Hoechst-stained promastigotes at 72 h as assessed by fluorescence microscopy depicting parasite death on glycoside treatment. (ii) Percentage PI positivity vs. percentage Hoechst positivity represents death vs. viability of promastigotes at 72 h against treatment by novel glycosides. **(D)** Cytotoxic effect of **Gly2** on clinical PKDL isolate BS12 in a concentration-dependent manner.

Glycoside derivatives are highly abundant in nature and have relatively simpler conformations due to which they are gaining current momentum as bioactive molecules in pharmaceutical industries (Prassas and Diamandis, 2008). Herein, the commercially available D-glucose 1 and N-Acetyl-D-glucosamine 2 molecules were coupled along with various short- to long-chain alcohols under acidic conditions to design and synthesize glycoside-derived compounds. The synthesis of ethyl glycoside *N*-Acetyl-D-glucosamine (Glycoside-1) was prepared by refluxing 1 in ethanol for 24 h in the presence of amberlite-H+ IR-120, which gave us the desired product Glycoside-1 as an anomeric mixture in good yield as described **(** **Figure 1A** **)**. The process does not involve any further purification, and amberlite resin can be reused by activating it with 1 N HCl in MeOH. In parallel, other compounds in this series, Glycoside-2 to Glycoside-6, were also prepared, adopting similar reaction protocols (methods), which resulted in good yields. Furthermore, the alkyl glycosides of N-Acetyl-D-Glucosamine Glycoside-7 to Glycoside-12 were prepared by refluxing N-Acetyl-D-Glucosamine with corresponding alkyl alcohols for 24 h in the presence of amberlite-H+ 100 IR-120 resin, which gave the desired products as an anomeric mixture in good yield as shown **(** **Supplementary Table S1** **).** Further characterizations of all 12 compounds were done using NMR and quadrupole/Q-TOF mass spectrometer with an ESI source determined using HRMS spectra **(** **Supplementary Material** **)**.

### Absorption, Distribution, Metabolism, and Excretion (ADMET) and Drug-Likeness Evaluation

SwissADME provides detailed and extensive physicochemical profiles and medicinal chemistry properties of any compound using five different algorithms (Daina et al., 2017). The partition coefficient and solubility are the two parameters that play important roles in the physicochemical aspect. So, based on predicted LogP value for the compounds that represents partition coefficient, it is concluded that all 12 compounds lie within the range value of 1.6–3.6, depicting the drug-likeness (Waring, 2010).

### *In Vitro* Metabolic Viability Assay-Based Screening Unraveled the Lead Glycoside Derivatives and Their Toxic Impact on *L. donovani* Promastigotes

To evaluate the cytotoxic effects of 12 compounds on *L. donovani* promastigotes, LDH assay was performed. This assay involves the reduction of tetrazolium salts to formazan during LDH-mediated catalysis of lactate to pyruvate, which can be detected at 490 nM. Rate of formazan formation is proportional to the release of LDH through damaged cell membranes. For the preliminary screening, the promastigotes were incubated with 12 compounds at a single concentration of 5 µM for a period of 72 h, and the released LDH was estimated **(** **Supplementary Figure S1A** **)**. Amphotericin B-treated promastigotes were taken as the positive control. A significant amount of formazan formation was observed in **Glycoside (Gly) 2-, 6-,** and **8**-treated samples, suggesting that these glycosides have a toxic effect on parasites. It was observed that **Gly2** (IC50 = 1.13 µM) demonstrated the lowest IC50 value as compared to **Gly6** (IC50 = 2.94 µM) and **Gly8** (IC50 = 1.61 µM), and hence, it has the most prominent lethal effect **(** **Figures 1Bi–iii** **)**. Having observed the antileishmanial effect of **Gly2** on growth and proliferation of promastigotes, we next investigated the cytotoxicity of **Gly2** on mammalian cells (MDCK, J774.1A) through MTT (3-(4, 5-dimethylthiazolyl-2)-2, 5-diphenyltetrazolium bromide) cell viability assay **(** **Supplementary Figures S1B, C** **)**. A minimum level of cytotoxicity could be detected for the compound in MDCK cells and macrophage cells. The CC50 values of **Gly2** analog was in the micromolar range. The selectivity index (SI) for the compound was calculated as the ratio between cytotoxicity (CC50) and activity (IC50) against promastigotes **(** **Table 1** **)**. The SI value for **Gly2** were more than 1,000, suggesting that the analogs are at least 1,000 times more specific to parasites than host cells. The above result suggests that **Gly2** imposed profound cytotoxic effects in Leishmania with no lethal effect in mammalian macrophages and canine epithelial MDCK, ensuring their specificity toward *L. donovani* promastigotes and not on the host.

**Table 1:** IC50/CC50/SI values of glycoside derivatives.

	IC50 (µM)	CC50 (µM) Macrophages	SI Macrophages
Gly1	65	247.9	3.813
Gly2	1.13	2,900	2,566.371
Gly3	12.7	1,677.6	132.090
Gly4	4.6	167	36.304
Gly5	5.97	159.2	26.666
Gly6	3.4	1,033	303.823
Gly7	77	299	3.883
Gly8	1.6	959	599.375
Gly9	36	256	7.111
Gly10	25.22	239	9.476
Gly11	43	179	4.162
Gly12	95	279	2.936

Promastigote death was further confirmed against **Gly2, 6,** and **8** treatment for a period of 72 h using live/dead dual staining of treated parasites with Hoechst/PI staining, respectively, and analyzed through fluorescent microscopy. The results revealed that untreated promastigotes showed viability (**Hoechst^+^/PI^-^**), whereas the treated parasites showed cellular death (**Hoechst^-^/PI^+^**) **(** **Figure 1Ci** **)**. Precisely, we found ~94.15% dead parasites in Gly2-treated samples as compared to ~33.72% and 75.47% in Gly6- and Gly8-treated promastigotes, respectively **(** **Figures 1Ci, ii** **)**.

We further evaluated the effect the **Gly2** on the clinical isolate of PKDL strain, BS12 of *L. donovani* (Sengupta et al., 2019). The results showed a pronounced toxic effect of **Gly2** on the Indian origin *Leishmania* isolate of PKDL. As mentioned previously, LDH assay was carried out on promastigotes treated with different concentrations of **Gly2** for 72 h, while amphotericin B was used a positive control. The promastigotes showed IC50 value at 1.97 µM with 50% parasitic death **(** **Figure 1D** **)**. Along with **Gly2**, we also evaluated the cytotoxic effects of 12 compounds on *L. donovani* promastigotes derived from clinical isolates of PKDL strain at 5 µM **(** **Supplementary Figure S2** **)**. The data strongly suggest that **Gly2** could adversely affect the metabolic cell viability of the lab strain of *L. donovani* along with the clinical strain of PKDL.

### Gly2 Inhibitor Has High Binding Affinity for *L. donovani* Gp63, A Promastigote Surface Molecule

**Figure 2:**
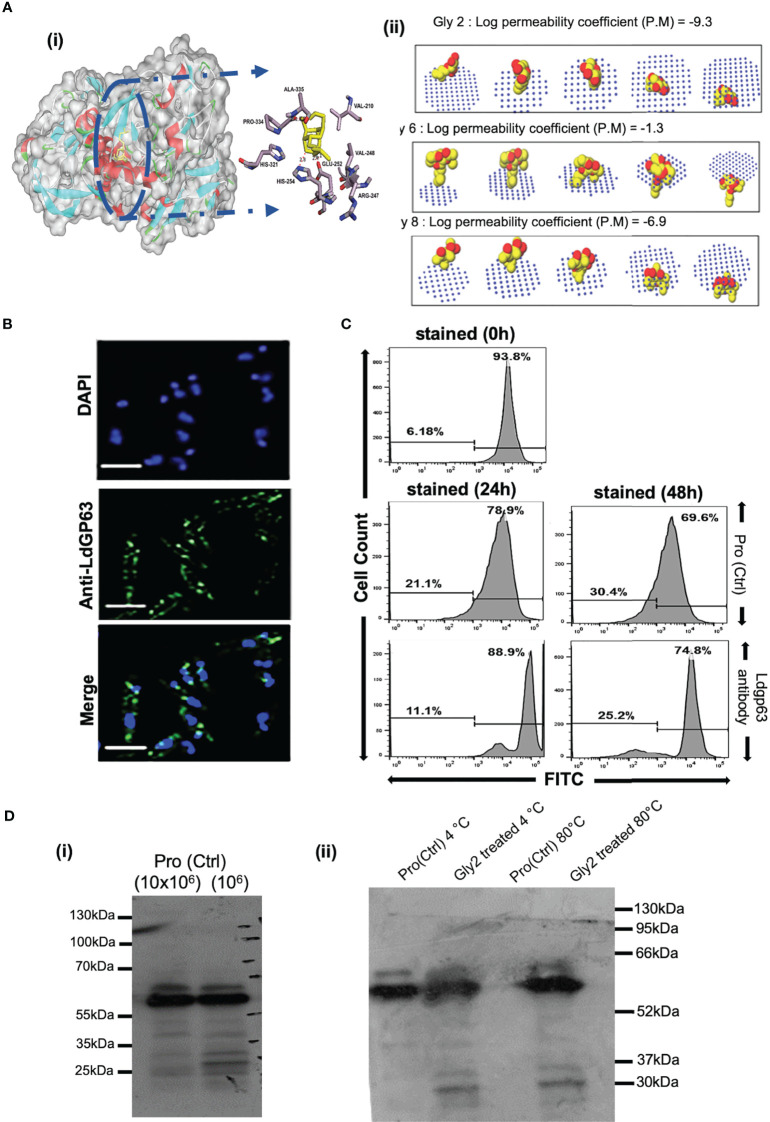
Gp63, a surface molecule, is the target of **Gly2** and is involved in promastigote proliferation. **(A)** (i) Three-dimensional (3D) surface model of Gly2–LdGp63 complex denoted by the degree of hydrophobicity and surface accessible for ligand binding using a gray scheme. The defined region shows interacting residues of Gly2–LdGp63 complex. Ligplot version 2.2, Discovery Studio version 19.1.0, and Pymol version 2.3.2 have been used to generate these figures. (ii) Membrane binding affinity of Glycosides 2, 6, and 8 using PerMM server. **(B)** Immunofluorescence assay of *Leishmania donovani* promastigotes using anti-LdGp63 antibody. **(C)** Proliferation of promastigotes determined by FACS masked with anti-LdGp63 antibody using CFDA-SE dye for a consecutive period of 24, 48, and 72 h. **(D)** (i) LdGp63 was detected in variable numbers of promastigote lysate with generated polyclonal anti-LdGp63 antibody. (ii) The thermostability of LdGp63 was analyzed by immunoblotting in untreated or **Gly2** (5 µM)-treated promastigotes at 4°C and 80°C.

Among the various molecules of *Leishmania* that are considered potential targets for drug development, Gp63 protein is the most potent surface target, as it is widely distributed over the entire surface of the promastigote. Gp63 has various functions in both the promastigote form and the amastigote form in mammalian hosts (Medina-Acosta et al., 1993). Since Gly2 enforced strong cytotoxic effects on both promastigotes of lab and clinical strain, leading to parasitic death, we hypothesized that the antileishmanial effect of Gly2 might be the result of Gly2–Gp63 interaction. As a proof of concept, we generated the structure of LdGp63 through homology modeling with *L. major* leishmanolysin in complex Zinc ion (1LML) as template that showed significant structural identity **(** **Supplementary Figure S3** **)**. The generated model of LdGp63 was further refined and analyzed for interaction with **Gly2, Gly6,** and **Gly8** through *in silico* docking. The best conformations of the docked compounds were selected based on their lowest free binding energy to the catalytic domain. Analysis of this pocket unraveled three residues (**His251, Glu252,** and **Pro334**) that are involved in binding to the compounds **(** **Figure 2Ai** **). Gly2** showed lower free binding energy as compared to others, suggesting efficient docking **(** **Supplementary Table S2** **)**. In the docked complex, the O3, O4, and O5 residues of Gly2 formed a strong H-bond interaction with **His251, Glu252,** and **Pro334** residues of LdGp63 (2.83Å, 2.71 Å, and 2.90 Å), respectively **(** **Figure 2Ai** **)**.

Since Gp63 is the surface molecule, we next investigated the passive permeability of **Gly2, Gly6,** and **Gly8** inhibitors across the lipid bilayer *in silico*. We found that **Gly2** was impermeable to the plasma membrane with log of permeability coefficient of -9.3 at temperature 310K and neutral pH of 7. On the other hand, **Gly6** was permeable to plasma membrane with log of permeability coefficient of -1.3. In comparison of **Gly2**, **Gly8** was impermeable to membrane with log of permeability coefficient of -6.9. Further using GLMol, the interactive 3D images of a compound moving across the membrane have been visualized **(** **Figure 2Aii** **)**. This strengthened our hypothesis regarding probable interaction of **Gly2** with membrane protein of parasite, leading to suitable target identification. Based on the docking result (interaction of **Gly2** with Gp63 catalytic domain), we have raised the antibody against the catalytic domain of Gp63 (LdGp63). The immunofluorescence assay with the LdGp63 antibody validated the maximum expression of Gp63 on the surface of the promastigote, whereas the rest remained localized to the intracellular level **(** **Figure 2B** **)**.

Due to surface abundance Gp63 in promastigotes and considering the functional diversity of Gp63, we speculated that masking the promastigote surface with catalytic domain-specific anti-LdGp63 antibody should affect the proliferation of promastigotes as well. To test this possibility, we stained the LdGp63 antibody-treated promastigotes with CFDA-SE, a cell-permeable dye. The decrease in fluorescence intensity is proportional to multiplication of the promastigote daughter cells (Messaritakis et al., 2010). The findings indicated subsequent changes in the percentage of CFDA-positive cells in LdGp63 antibody-treated parasites for 24 (88.9%) and 48 h (74.8%) as compared to untreated cells at 24 (78.9%) and 48 h (69.6%), respectively **(** **Figure 2C** **)**.

Furthermore, to verify the *in vitro* binding efficacy of **Gly2** inhibitor with gp63 in a physiologically relevant cellular environment, i.e., promastigote lysates, we performed **CETSA**. For this, first, the number of promastigotes (10^4^–10^7^ cells) was titrated and immunoblotted with LdGp63 antibody **(** **Figure 2Di** **)**. We then focused on **Gly2-**treated and untreated promastigote lysates (10^7^ cells), which were incubated for 24 h and subsequently exposed to different temperatures before immunoblotting. The results of the immunoblot demonstrated that, at higher temperature (80°C), the Gp63 molecule in the untreated cell lysate is destabilized, while in lysates treated with **Gly2,** Gp63 is found to be stable at higher temperature **(** **Figure 2Dii** **)**. Here, the binding of **Gly2** to Gp63 caused protein stabilization event under physiologic conditions even at a remarkably high temperature of 80°C, without any sign of denaturation. This clearly suggested ligand-dependent stabilization of Gp63 by **Gly2** even at a much higher temperature than the normal physiological temperature, strongly corroborating *in silico* and *in vitro* analyses that represented **Gly2** as the potent inhibitor of Gp63.

### Anti-Proliferative Effect of Gly2 Is Due to Disruption of Mitochondrial Membrane and Destabilization of Redox Potential

**Figure 3:**
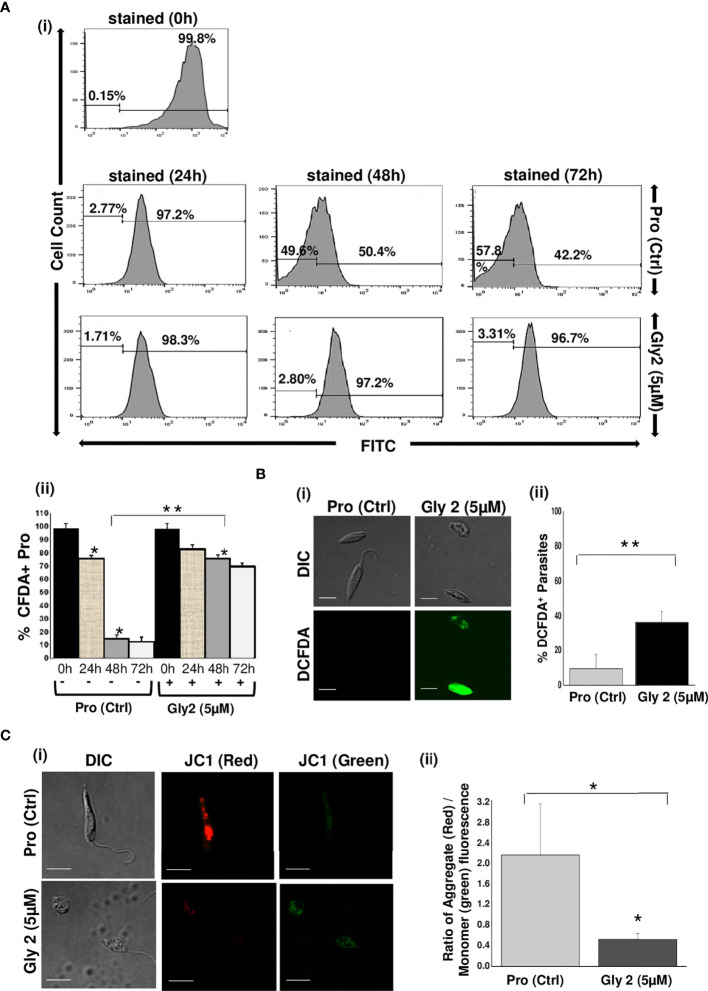
Gly2 efficiently reduced promastigote proliferation, leading to elevated ROS levels and disruption of mitochondrial membrane potential. **(A)** (i) Rate of proliferation of promastigotes was determined by the change in CFDA-SE staining as represented by flow cytometry histograms, depicting huge cellular multiplication arrest upon treatment with Gly2 at 24, 48, and 72 h, respectively. Pro refers to untreated promastigotes. (ii) Nominal decrement observed in the percentage of CFDA-SE-positive promastigotes when treated by the compound for 24, 48, and 72 h. The asterisks (**) indicate statistical significance (p < 0.01, n = 3) between the indicated groups. **(B)** (i) Confocal imaging of DCFDA staining showed strong green fluorescence in Gly2-treated cells indicative of increased ROS levels. (ii) Bar graph representing significantly higher percentage of DCFDA+ promastigotes in Gly 2 treatment as compared to control. **(C)** (i) Effect of Gly2 on ΔΨm of promastigotes was indicated by the conversion of monomer (green) to oligomer (red) forms of JC-1 using confocal micrographs. The shift in intensity of red fluorescence (JC1 aggregates/PE) to green fluorescence (JC1 monomers/FITC) implies destabilized ΔΨm in promastigotes following the treatment. (ii) Bar graph showing the reduction in the ratio of JC-1 Red (aggregate)/Green (monomer) in Gly2-treated parasites. The asterisks (* and **) indicate statistical significance (p < 0.05 and p < 0.01, respectively, n = 3) between the indicated groups.

It has been reported that downregulation of Gp63 protein results in the loss of promastigote development and multiplication (Hajmová et al., 2004; Pandey et al., 2004). Based on this information, we hypothesized that the binding of **Gly2** to Gp63 protein might have an anti-proliferative effect on promastigotes. Therefore, we assessed the proliferation of promastigotes by quantifying the release of CFDA-SE, a cell-permeable dye, during cell division. It was observed that, when the promastigotes were treated with 5 µM of **Gly2**, the percentage of CFDA-positive cells remained unchanged at 24 (81.59%), 48 (74.68%), and 72 h (68.48%) that implied the absence of cell division in the parental cell. However, the untreated promastigotes showed proliferation in a time-dependent manner as the CFDA-positive cells reduced gradually from 24 to 72 h **(** **Figure 3A** **)**. From the above results, we deduced that there is a significant reduction in promastigotes’ growth and multiplication upon treatment with **Gly2.** Previous studies, including the study from our own lab, have shown that the cellular mechanism of antileishmanial drugs might involve destabilization of ΔΨm-coupled ROS elevation leading to cellular death patterns in *Leishmania.* spp. (Corral et al., 2016; Ramu et al., 2017). Therefore, we then asked whether **Gly2**-based inhibition of LdGp63 has any similar impact. To address the same, we have used DCFDA-based detection of ROS levels in promastigote forms of *L. donovani* (Corral et al., 2016; Ramu et al., 2017). The data represented an increased intensity of green fluorescence in **Gly2**-treated promastigotes, indicating enhancement in intracellular ROS levels, whereas control parasites showed balanced redox homeostasis **(** **Figure 3B** **)**. The representative bar graph showed ~50% parasite population with DCFDA staining (% DCFDA**^+^** parasites) suggestive of elevated ROS levels **(** **Figure 3B** **)**. Elevation of intracellular ROS is usually coupled with the depolarization of mitochondrial membrane. Hence, to explore the effect of **Gly2** on ΔΨm of promastigotes, we have used a lipophilic cationic dye (JC-1) exhibiting green fluorescence in its monomeric form. Enhanced level of red fluorescence denotes more J aggregate formation due to higher ΔΨm, whereas a shift toward lower red and/or accumulation of higher green fluorescence implies a strong indication of destabilized ΔΨm (Sivandzade et al., 2019). Upon treatment of promastigotes with **Gly2**, we found that the mitochondrial uptake of JC-1 dye was significantly decreased, as manifested by stronger green fluorescence as compared to the control healthy promastigotes displaying intense red fluorescence, depicting stable ΔΨm **(** **Figure 3C** **)**. These data clearly inferred that in addition to its anti-proliferative effect, **Gly2** elevated the intracellular levels of ROS, leading to loss of ΔΨm.

### Gly2 Treatment Demonstrated a Significant Reduction in Infected Macrophages and Intramacrophagic Form of Amastigotes

**Figure 4:**
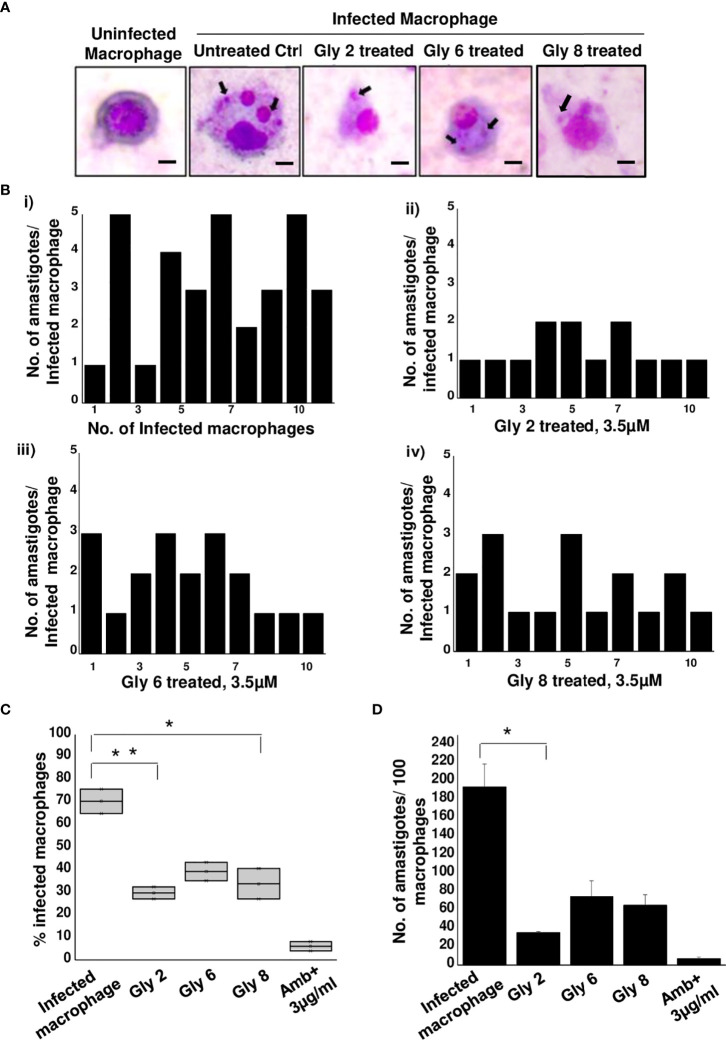
Antileishmanial activity of **Gly2** against intramacrophage amastigotes. **(A)** Glycoside-treated and untreated Geimsa-stained mouse macrophages infected with *L. donovani* amastigotes. **(B)** Number of amastigotes per infected macrophage in untreated (i) and glycoside (ii–iv)-treated cells that were counted individually for 10 distinct cells. **(C)** Percentage of infected macrophages in the presence and absence of glycoside treatment. **(D)** Number of amastigotes per 100 macrophages in both untreated and glycoside-treated cells. P-values *<0.05 and **<0.01 were considered as values denoting significance.

With promising antileishmanial effect of Gly2 on promastigote forms of *Leishmania* spp., we then studied its impact on intramacrophagic amastigote forms of *Leishmania*. The findings revealed that the parasite infection is severely reduced following glycoside treatment **(** **Figure 4A** **)**. Furthermore, we asked whether these treatments could also affect the number of amastigotes per infected macrophage. As expected, the number of amastigotes was found to be predominantly reduced in **Gly2**-treated samples (~1 amastigotes/macrophages), wherein **Gly6** and **Gly8** treatments depicted ~3 or 2 amastigotes/macrophages, respectively **(** **Figures 4Bi–iv** **)**. The control untreated macrophages had ~5 numbers of amastigotes/macrophages. In addition, the percentage of macrophages infected was found to be very less following glycoside treatment as compared to untreated control **(** **Figure 4C** **)**. The total amastigote load was also calculated per 100 macrophages, and the results have shown severe depletion in total amastigote load against glycoside treatments as compared to control **(** **Figure 4D** **)**. Plausibly, **Gly2** has a pronounced effect on intracellular amastigotes in exceptionally low micromolar range, suggesting its potential antileishmanial activity in both stages.

### Gly2 Induced Enhancement of Complement-Mediated Lysis of Promastigotes in Serum Physiological Conditions

**Figure 5:**
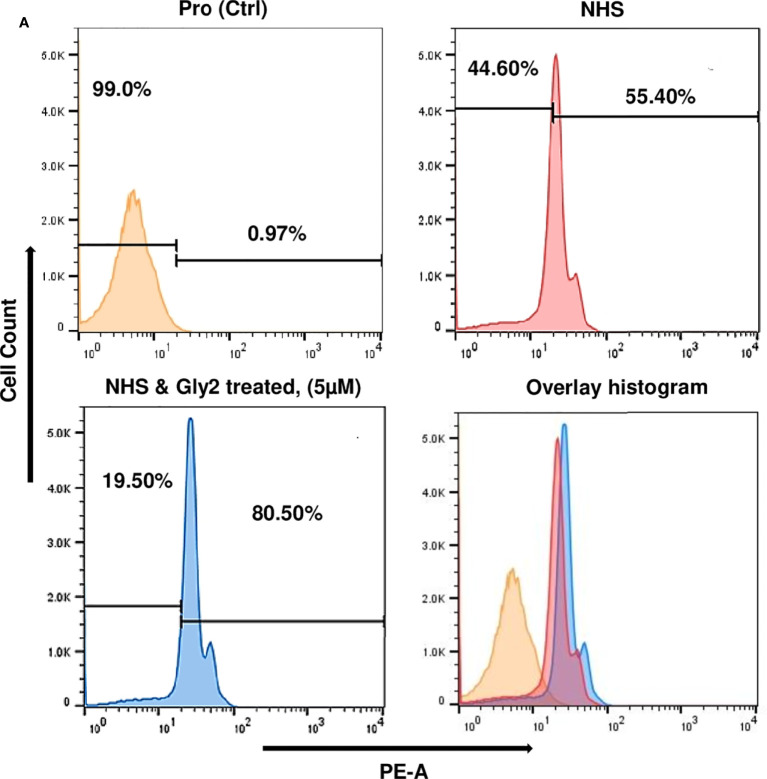
**Gly2**-treated promastigotes enhance the complement-mediated lysis in serum physiological condition and the concentration-dependent cytotoxic effect of Gly2 on PKDL strain. **(A)** Promastigote lysis, triggered by 10% normal human serum, was analyzed by uptake of PI using flow cytometry.

Mimicking the physiological condition of the body, axenic promastigote cultures in late log phase were exposed to complement-mediated lysis using human serum, which was determined by measuring PI-stained cells (Domínguez et al., 2002; Bandyopadhyay et al., 2004). Histogram plots illustrate 55.40% population of promastigote death in 10% NHS within 30 min as compared to control that showed 0.06% parasite death and 99.00% intact promastigotes in 10% FBS (heat inactivated). The **Gly2**-treated promastigotes however showed 80.05% parasite death in the presence of 10% NHS. There was 24.65% increment in PI uptake that is directly proportional to promastigote killing. Majority of **Gly2**-treated promastigotes were non-motile when observed under the microscope. The precise size of this population was difficult to calculate, as in the presence of NHS, promastigote cell volume and refractile properties were altered, blurring the distinction between promastigotes, cell debris, and NHS background signal. These results indicate that the presence of **Gly2** and 10% NHS causes rapid lysis of *Leishmania* promastigotes **(** **Figure 5**, **Supplementary Figure S4** **)**.

## Discussion

The major blockades involved in therapeutic interventions against leishmaniasis include inefficacious pharmacokinetics and pharmacodynamics of drugs, toxicity, drug resistance, and lack of vaccines (Sundar and Singh, 2018). These factors create an urgent requirement for discovering novel drug candidates and developing target-specific molecules for better preventive measures and treatment modalities. Earlier reports have elucidated the important role of flavonoid glycosides from biologically active aqueous plant extracts against VL (Shah et al., 2014). Notably, plant-based beta-glycosides and their aglycones have been detected as potent transmission-blocking sugar baits that alter the physiology of the vector *L. longipalpis* and the development of *Leishmania* spp. inside the same (Ferreira et al., 2018). Based on these facts, we have synthesized simple glucose and glucosamine backbone conferring natural product-inspired library of compounds using green synthetic chemistry with strong compliance to Lipinski’s “rule-of-five.” To evaluate the cytotoxic effect of these glycoside derivatives, on both parasites and host, we performed *in vitro* screening of the same on *L. donovani* promastigotes and in mammalian cells such as macrophages and MDCK. The findings represented profound cytotoxicity conferred by **Gly2, 6,** and **8** on promastigotes, leading to significant parasitic death, with no lethal impact on mammalian cells. These data suggested strong selectivity of compounds toward parasites, also clearly depicted by their representative higher SI. However, among these compounds, Gly2 demonstrated the highest SI value (>1,000) and enhanced toxicity in both lab strain of *L. donovani* and in BS12, a clinical strain of PKDL, suggesting its potent antileishmanial ability.

Considering the profound cytotoxic effects enforced by **Gly2** on both lab and clinical forms of promastigotes, we assumed that Gly2 might possibly interact with membrane-associated proteins. It is noteworthy that Gp63/Leishmanolysin, a metalloproteinase containing a conserved zinc-binding/catalytic motif (HEXXH), is abundantly expressed on the surface of promastigotes and known to play significant roles in multiple steps of the infection comprising their entry into macrophages until their intralysosomal survival (Liu and Chang, 1992; Brittingham et al., 1999; Cerdà-Costa and Gomis-Rüth, 2014). Since LdGp63 is widely expressed on the surface of promastigotes, it is hypothesized that the antileishmanial effect of Gly2 might be the result of Gly2–Gp63 interaction. Further *in silico* docking analysis of the 3D surface model of Gly2–LdGp63 complex revealed efficient binding of Gly2 with a unique pocket in the catalytic domain of LdGp63, strengthening this hypothesis. Next, using an *in silico* tool, the membrane permeability aspect of **Gly2, Gly6, and Gly8** was assessed that clearly depicted Gly2 to be the only non-permeant glycoside derivative with a strong membrane binding affinity, suggestive of its strong interaction with the catalytic pocket of LdGp63 expressed on the promastigote surface. This finding further enabled us to have a deeper look at the chemical structures of lead compounds, which revealed that **Gly2,** the most active compound derived from D-glucose, has isopentyl as branched alkyl group with native free hydroxyl (OH) group that makes this molecule hydrophilic (clogP 0.26) and less cell penetrable. **Gly6,** having isopentyl as branched alkyl chain along with OH group, is being masked with acetyl (OAc) group, which makes the molecule more hydrophobic (clogP 2.4) that is suitable for cell permeability. **Gly8**, on the other hand, has a shorter straight or linear alkyl chain (propyl) along with masked OH group, representing a potent amphiphile (clogP 1.47) that might perform dual roles including surface binding and cell penetration. Plausibly, **Gly2**-mediated abrogation of promastigote multiplication further strengthened the possibility of Gly2–LdGp63 interaction on the promastigote surface. In addition, masking of promastigote surface with in-house-generated catalytic domain-specific mouse polyclonal anti-LdGp63 antibody represented partial abrogation of promastigote proliferation, substantiating the role of LdGp63 in proliferation of promastigotes. Furthermore, to assess interactions between **Gly2** and LdGp63 in a physiologically relevant cellular environment, we performed CETSA that has emerged as an efficient tool to validate drug–target engagement and target validation both in complex protein samples and in live cells. Precisely, CETSA solely works upon the principle of ligand binding-induced thermal stabilization of proteins. This assay induces heat-induced denaturation and precipitation of unbound proteins when subjected to higher temperatures, leaving the ligand-bound proteins in the solution that can be detected by Western blot (Langebäck et al., 2019). An accumulating number of studies suggest that CETSA has been widely used as a primary validation of drug–target engagement in case of apicomplexan parasites such as *Plasmodium falciparum* and *Toxoplasma gondii* (Herneisen et al., 2020).

In this study, CETSA revealed that Gly2 upon binding with Gp63 displayed protein complex stabilization even at a remarkably high temperature of 80°C under physiologic conditions. This phenomenon could be visualized prominently when the membrane was immunoblotted with anti-LdGp63 catalytic domain antibody, which represented an abundance of Gp63 in treated samples, confirming the possibility of high affinity binding of **Gly2** to LdGp63. Previous studies, including one from our group, have shown that drug treatment has a core impact toward parasite death through loss of ΔΨm and ROS elevation, a coupled event associated with cellular death patterns in *Leishmania* spp. (Ramu et al., 2017). In line with such information, we checked if **Gly2** has any such impact on *L. donovani* promastigotes. As assumed, the results confirmed increased ROS generation followed by ΔΨm loss, leading to almost ~90% of parasitic death against **Gly2** treatment. With such profound impact on promastigotes, next, we asked whether **Gly2** could impose any changes to intramacrophagic amastigote forms of *L. donovani*. To highlight, **Gly2** treatment indeed demonstrated significant depletion in infected macrophages and led to a substantial reduction in load of intracellular amastigotes as compared to the other glycoside hybrids. It is noteworthy that *Leishmania* modulates the complement system for its survival in the host, and the resistance to such innate immune component is associated with its two major GPI-anchored surface glycoconjugates, namely, lipophosphoglycan (LPG) and GP63 (Cecílio et al., 2014; Verma et al., 2018; Elmahallawy et al., 2021). Interestingly, *L. major*, being deficient with these two surface determinants, has shown high sensitivity toward the complement system (Joshi et al., 2002; Späth et al., 2003). Thus, based on such information, we have determined the impact of Gly2 treatment on susceptibility of promastigotes toward complement-mediated lysis in NHS-enriched promastigote culture that mimicked similar pathophysiological conditions in the body. The observations suggested prominent increment in parasitic death contributing to ~80% of cytolysis. Overall, our study has discovered Gly2 as a novel glycoside hybrid with promising antileishmanial activity.

## Conclusions

In the current scenario of drug-resistant *Leishmania* parasites, there is a requirement to develop new potent antileishmanials that are less prone to resistance development. Conclusively, we have introduced a novel glycoside derivative, **Gly2**, as a potential antileishmanial and suggested a prospective first-in-class therapeutic measure for both VL and PKDL to ascertain its clinical utility.

## Data Availability Statement

The original contributions presented in the study are included in the article/**Supplementary Material**, further inquiries can be directed to the corresponding authors.

## Author Contributions

AC has contributed to performing all the biological experiments. RS & CN designed the molecules and CN synthesized the molecules.. AC and NJ have done preliminary analyses and *in silico* membrane binding studies and prepared the initial draft. SS and SP have conceived the study and planned the work plan. SG and SP have executed, troubleshooted, and analyzed the data. AR and LG have performed data analyses. AC and SP have written the final draft. SS, SP, and RS have corrected and edited the final version of the article.

## Funding

This work was supported by funding from DBT builder program, DST, and JNU UPE II program. Dr. Shailja Singh and Dr. Anand Ranganathan are thankful for the funding support from the Science and Engineering Research Board (SERB, File no. IPA/2020/000007) and Drug and Pharmaceuticals Research Programme (DPRP, Project No. P/569/2016-1/TDT). Dr. Soumya Pati is grateful for the funding support from the Cognitive Science Research Initiative (CSRI) program of the Department of Science and Technology (DST/CSRI/2018/247).

## Conflict of Interest

The authors declare that the research was conducted in the absence of any commercial or financial relationships that could be construed as a potential conflict of interest.

## Publisher’s Note

All claims expressed in this article are solely those of the authors and do not necessarily represent those of their affiliated organizations, or those of the publisher, the editors and the reviewers. Any product that may be evaluated in this article, or claim that may be made by its manufacturer, is not guaranteed or endorsed by the publisher.
