# Supplementary material for: Metalloprotease Gp63-Targeting Novel Glycoside Exhibits Potential Antileishmanial Activity
Source: Front Cell Infect Microbiol. 2022 May 4;12:803048. doi: 10.3389/fcimb.2022.803048 (PMC9115111; doi:10.3389/fcimb.2022.803048)

**Metalloprotease Gp63 targeting novel glycoside exhibits potential antileishmanial activity**

Amrita Chakrabarti^1^**^§^**, Chintam Narayana^2^**^§^**, Nishant Joshi^1^, Swati Garg^1,3^, Lalit Garg^4^, Anand Ranganathan^3^  , Ram Sagar^2,5,6^**^*^**, Soumya Pati^1^**^*^**, Shailja Singh^1,3^ **^*^**

*^1^Department of Life Sciences, School of Natural Sciences, Shiv Nadar University, Uttar Pradesh 201314, India*

*^2^Department of Chemistry, School of Natural Sciences, Shiv Nadar University, Uttar Pradesh 201314, India*

*^3^Special Centre for Molecular Medicine, Jawaharlal Nehru University, New Delhi, India*

*^4^Gene Regulation Laboratory, National Institute of Immunology, New Delhi, India*

*^5^Department of Chemistry, Institute of Science, Banaras Hindu University, Varanasi 221005, Uttar Pradesh, India*

*^6^School of Physical Sciences, Jawaharlal Nehru University (JNU) New Delhi 110067, India.*

^*^*Email:* [*shailja.jnu@gmail.com*](mailto:shailja.jnu@gmail.com)*,* [*soumya.pati@snu.edu.in*](mailto:soumya.pati@snu.edu.in)*,* *ram.sagar@mail.jnu.ac.in*

§ These authors contributed equally to this work.

**Supplementary Table 1: The structures of all the glycosides (1-12) are depicted.**


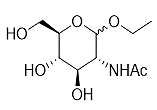

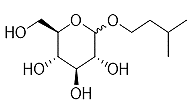

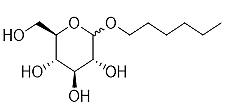


**Gly 1**

**Gly 2**

**Gly 3**


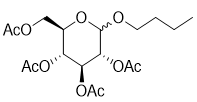

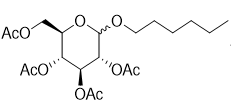

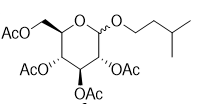


**Gly 4**

**Gly 5**

**Gly 6**


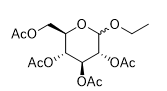

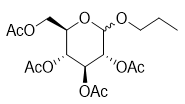

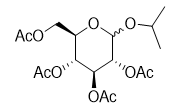


**Gly 7**

**Gly 8**

**Gly 9**


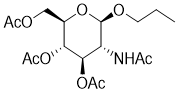


**Gly 10**


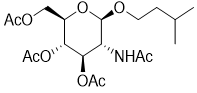


**Gly 11**


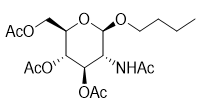


**Gly 12**


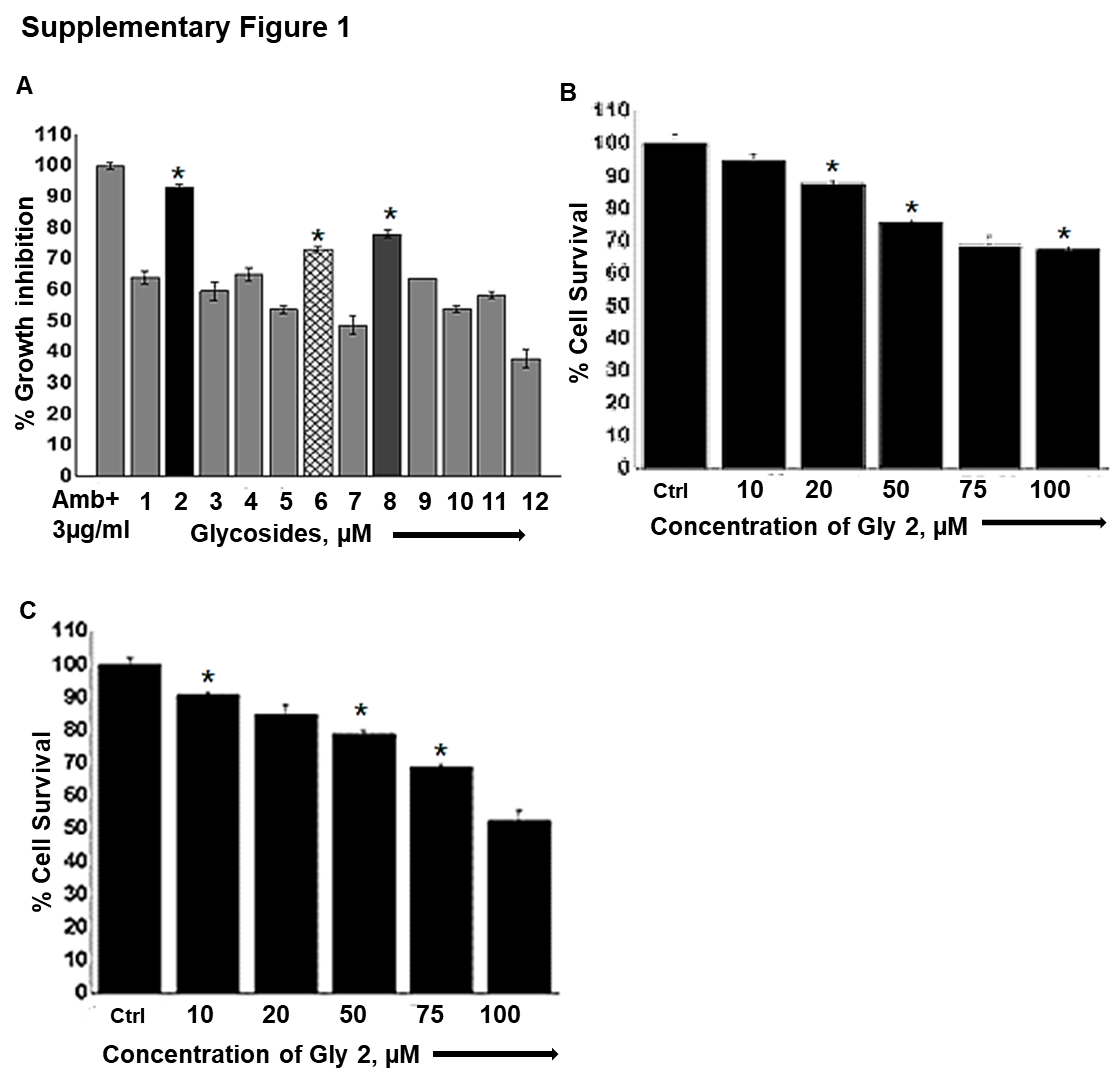


**Supplementary Figure 1:** *In vitro* screening of glycosides against promastigotes of Ag83 strain of *Leishmania donovani* to identify the lead compound. A) All the synthesized glycosides (1-12) were screened at 5µM concentrations against promastigotes of Ag83 strain; B-C) Cytotoxic effect of Gly 2 on MDCK and mouse macrophages J774.1A in concentration dependent manner using MTT Assay.

**Supplementary Figure 2**


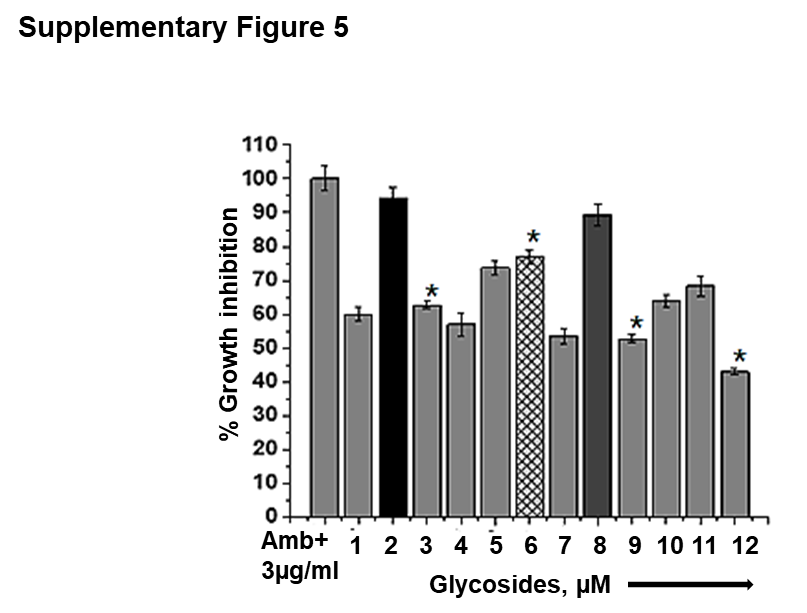


**Supplementary Figure 2:** *In vitro* screening of glycosides against promastigotes of clinical isolate of PKDL *Leishmania donovani* to identify the lead compound.

**Supplementary Figure 3**

**Supplementary Figure 3:** *In silico* Superimposition of LdGp63 and LmGp63 models showed significant structure identity.


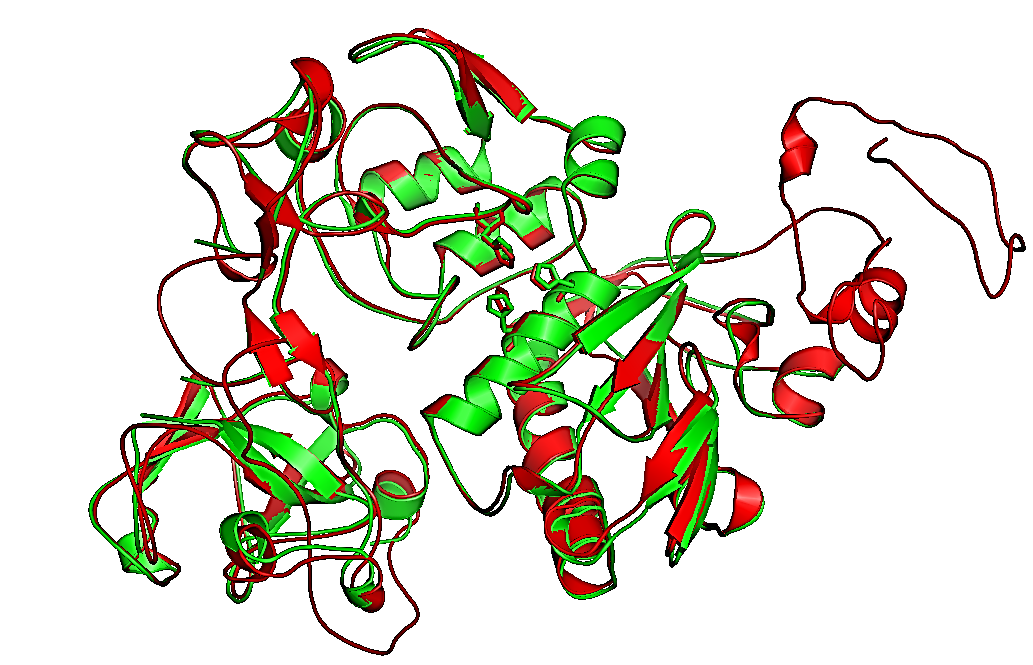


***L. donovani* Gp63**

***L. major* Gp63**

**Supplementary Figure 4**

**
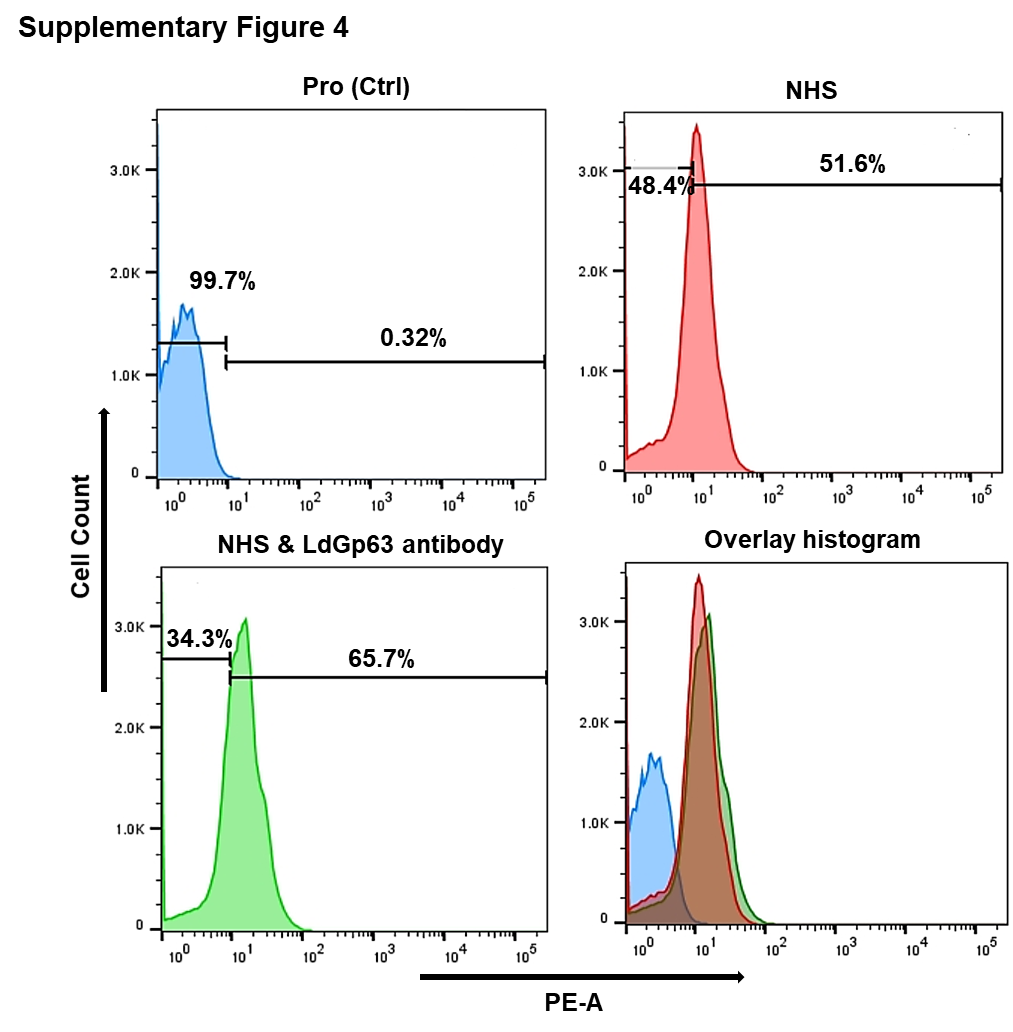
**

**Supplementary Figure 4:** Promastigote lysis was analysed by uptake of PI using flow cytometry triggered by 10% normal human serum (NHS) and Anti-LdGp63 antibody as compared to controls.

**Supplementary Table 2: Critical residues lying within the docked ligand-protein complex were found to be forming H-bonds with highest minimum binding energy with Gly 2.**

**
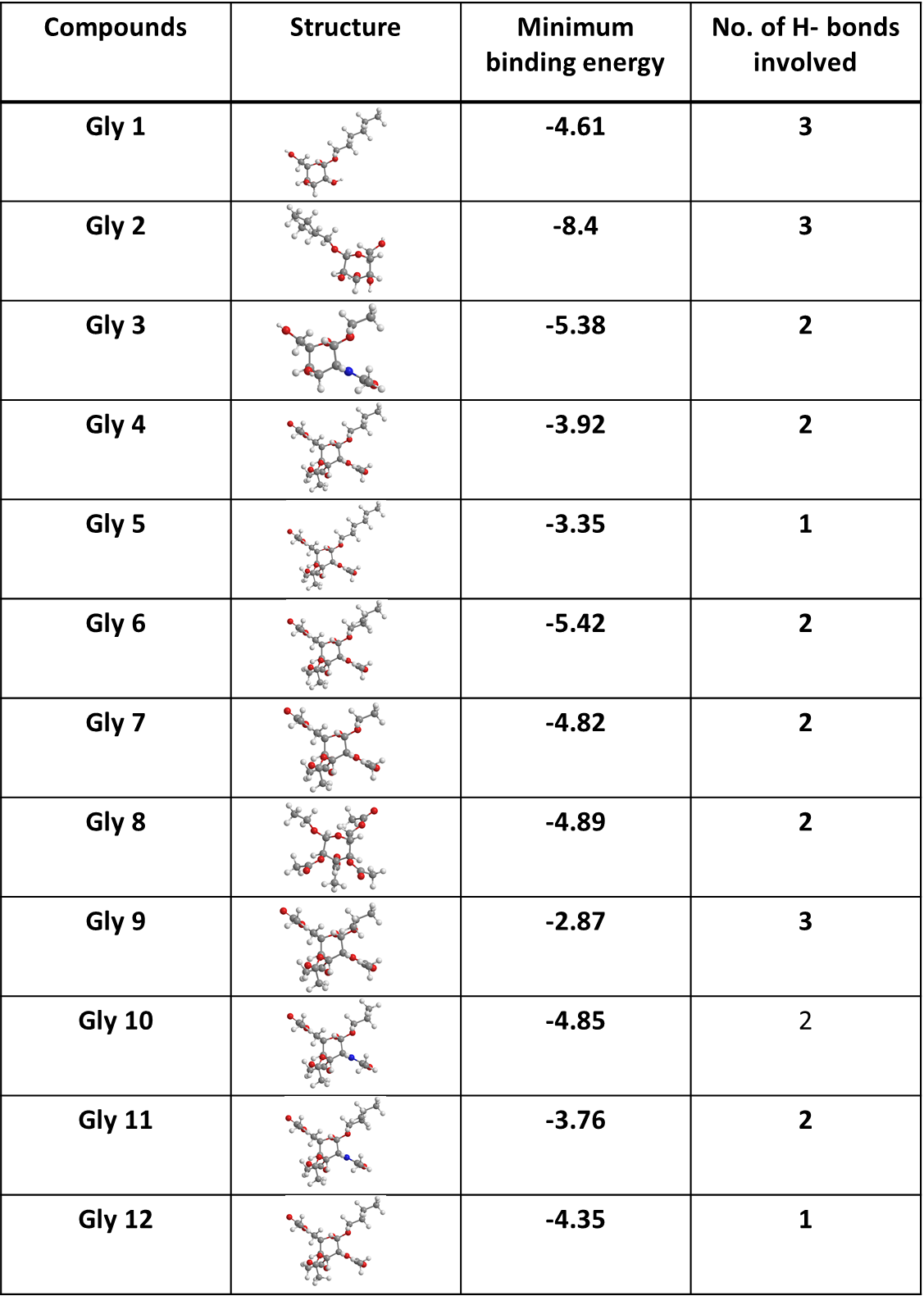
**

Supplementary Text

Characterization of glycoside derivatives by NMR analysis.

*N*-((3*R*,4*R*,5*S*,6*R*)-2-ethoxy-4,5-dihydroxy-6-(hydroxymethyl)tetrahydro-2*H*-pyran-3-yl) acetamide [Gly 1] : The title compound was isolated by column chromatography (EtOAc/MeOH = 95:5) in 41% yield. Compound was white color solid; anomeric ratio (α:β, 9:1)^: 1^H NMR (400 MHz, D_2_O): δ 5.21 (d, *J* = 3.4 Hz, 0.14H, H-1α), 4.89 (d, *J* = 3.5 Hz, 1H, H-1β), 3.92-3.85 (m, 3H), 3.81-3.71 (m, 4H), 3.55-3.46 (m, 3H), 2.05 (s, 4H), 1.20 (t, *J* = 7.2 Hz, 3H); ^13^C NMR (100 MHz, D_2_O): δ 174.4 (C=O), 96.6 (C-1β), 71.8, 71.1, 70.1, 64.0, 60.6, 53.7, 21.9, 14.0, HRMS(ESI) *m/z* Calcd for C_10_H_19_NO_6_, [M+Na]^+^ 272.1105, found 272.1139.

**(2*R*,3*S*,4*S*,5*R*)-2-(hydroxymethyl)-6-(isopentyloxy)tetrahydro-2H-pyran-3,4,5-triol [Gly 2]:**The title compound was isolated by column chromatography (EtOAc/MeOH = 95:5) furnished compound 2 as sticky solid 0.12 g (44 %), anomeric ratio (α: β = 1:6); ^1^HNMR (400 MHz, D_2_O) δ 4.91 (d, *J* = 4 Hz, 1H, H-1β), 4.45 (d, *J* = 8Hz, 0.3H), 4.00-3.87 (m, 1H), 3.84-3.77 (m, 1H), 3.74-3.67 (m, 3H), 3.58-3.52 (m, 2H), 3.48 (dd, *J* = 7.6 Hz, 1H), 3.40 (t, *J* = 9.6 Hz, 1H), 3.25 (t, *J* = 8.4 Hz, 1H), 1.74-1.68 (m, 1.3H), 1.61-1.46 (m, 2H), 0.92-0.90 (m, 7H); ^13^C NMR (100 MHz, D_2_O) 102.1 (C-1β), 98.0 ( C-1α), 75.9, 73.2, 71.8, 71.5, 71.3, 69.6, 69.1, 66.9, 60.8, 60.6, 37.7, 37., 34.2, 25.7, 24.4, 24.3, 22.1, 21.8, 21.7, 16.17, 10.7; HRMS (ESI) *m/z* Calcd for C_11_H_22_O_6_, [M+Na]^+^ : 273.1309, found 273.1342.

(3*R*,4*S*,5*S*,6*R*)-2-(hexyloxy)-6-(hydroxymethyl)tetrahydro-2H-pyran-3,4,5-triol [Gly 3] : The title compound was isolated by column chromatography (EtOAc/MeOH = 95:5) furnished compound 3 as yellow colour sticky solid 0.13 g (44 %), anomeric mixture (α:β, 2:1); ^1^HNMR (400 MHz, D_2_O) δ 4.91 (d, *J* = 4 Hz, 1H, H-1β), 4.46 (d, *J* = 8 Hz, 0.5H, H-1α), 3.94-3.90 (m, 1H), 3.85 (dd, *J* = 2 Hz, 12.4 Hz, 1H), 3.79-3.67 (m, 5H), 3.57-3.53 (m, 2H), 3.49 (t, *J* = 8.8 Hz, 1H), 3.43 (d, *J* = 9.2 Hz, 1H), 3.39 (dd, *J* = 6.8 Hz, 8.8 Hz, 1H), 3.26 (t, *J* = 6 Hz, 0.5H), 1.63 (quin, *J* = 6.4 Hz, 3H), 1.39-1.31 (m, 10H), 0.88 (t, *J* = 6.4 Hz, 4.8H); ^13^C NMR (100 MHz, D_2_O) 102.18 (C-1β), 98.08 ( C-1α), 75.9, 75.8, 73.1, 71.7, 71.3, 70.7, 69.6, 69.5, 68.3, 60.7, 60.52, 30.85, 30.82, 28.72, 28.58, 25.09, 24.75, 21.95, 21.92, 13.35, 13.32; HRMS(ESI) m/z Calcd for C_12_H_24_O_6_ [M+Na]^+^ 287.1465, found 287.1506.

(2R,3R,4S,5R)-2-(acetoxymethyl)-6-butoxytetrahydro-2H-pyran-3,4,5-triyltriacetate) [Gly 4] : The title compound was isolated by column chromatography (EtOAc/hexane = 1:9). furnished compound 6 as sticky solid 0.1 g in in 57 % yield; (α:β, 2:1): ^1^H NMR (400 MHz, CDCl_3_) δ ^1^H NMR (400 MHz, CDCl_3_) δ 5.44 (t, *J* = 9.8 Hz, 1H), 5.17 (t, *J* = 9.5 Hz, 0.5H), 5.08 – 5.00 (m, 2H), 4.95 (dd, *J* = 16.9, 8.8 Hz, 1H), 4.81 (dd, *J* = 10.2, 3.7 Hz, 1H), 4.45 (d, *J* = 8.0 Hz, 0.5H), 4.22 (dt, *J* = 12.2, 4.5 Hz, 1H), 4.13 – 4.08 (m, 1H), 4.06 (dd, *J* = 12.3, 2.1 Hz, 1H), 3.98 (ddd, *J* = 10.1, 4.4, 2.2 Hz, 1H), 3.84 (dt, *J* = 9.6, 6.3 Hz, 0.5 H), 3.68 – 3.62 (m, 2H), 3.47-3.37 (m, 2H), 2.05 (s, 3H), 2.04 (s, 1H), 2.02 (s, 3H), 2.00 (s, 1H), 1.99 (s, 3H), 1.98 (s, 1H), 1.97 (s, 3H), 1.96 (s, 1H), 1.57-1.51 (m, 3H), 1.38-1.30 (m, 3H), 0.91-0.84 (m, 5H).; ^13^C NMR (100 MHz, CDCl_3_) 170.74, 170.71, 170.3, 170.2, 170.1, 169.6, 169.4, 169.3, 100.9, 95.7, 72.9, 71.8, 71.4, 71.0, 70.3, 69.9, 68.7, 68.6, 68.5, 67.2, 62.1, 62.0, 31.4, 31.3, 20.77, 20.71, 20.68, 20.66, 19.2, 19.0, 13.8, 13.7. HRMS(ESI) *m/z* Calcd for C_18_H_28_O_10_, [M+NH_4_]^+^ 422.2021, found 422.2086.

(2R,3R,4S,5R)-2-(acetoxymethyl)-6-(hexyloxy) tetrahydro-2H-pyran-3,4,5-triyltriacetate) [Gly 5]: The title compound was isolated by column chromatography (EtOAc/Hexane = 1:9) furnished compound 6 as sticky solid 0.04 g in in 49 % yield; (α:β, 2:1) ^1^H NMR (400 MHz, CDCl_3_) δ 5.47 (t, *J* = 9.8 Hz, 3H), 5.19 (t, *J* = 9.5 Hz, 0.4 H), 5.09 – 5.04 (m, 2H), 5.09-4.95 (m, 0.6 H), 4.98 (dd, *J* = 17.1, 8.8 Hz, 2H), 4.87 – 4.80 (m, 3H), 4.48 (d, *J* = 7.9 Hz, 1H), 4.25 (dd, *J* = 12.2, 4.2 Hz, 1H), 4.14 - 4.06 (m, 1H), 4.01 (dd, *J* = 10.2, 2.4 Hz, 3H), 3.86 (dt, *J* = 9.4, 6.4 Hz, 0.3 H), 3.67 (dt, *J* = 9.5, 6.7 Hz, 4H), 3.48 - 3.38 (m, 1H), 2.08 (s, 3H), 2.07 (s, 1H), 2.02 (s, 1H), 2.019 (s, 3H), 2.01 (s, 1H), 2.00 (s, 3H), 1.99 (s, 1H),1.62-1.53 (m, 8H), 1.38-1.21 (m, 26H), 0.89 (t, *J* = 6.5 Hz, 12H).^13^C NMR (100 MHz, CDCl_3_) δ 170.8, 170.4, 170.3, 170.2, 169.7, 169.4, 100.9, 95.7, 73.0, 71.8, 71.5, 71.1, 70.4, 68.88, 68.80, 68.6, 67.2, 62.15, 62.10, 31.6, 29.4, 29.3, 25.8, 25.6, 22.7, 20.8, 20.79, 20.76, 14.1. HRMS(ESI) *m/z* Calcd for C_20_H_32_O_10_, [M+NH_4_]^+^ 450.2334, found 450.2411.

**(2R,3R,4S,5R)-2-(acetoxymethyl)-6-(isopentyloxy)tetrahydro-2H-pyran-3,4,5-triyltriacetate) [Gly 6]:**The title compound was isolated by column chromatography (EtOAc/Hexane = 10:90) furnished compound 5 as sticky solid 0.04 g (48 %), anomeric ratio (α: β = 2:1); ^1^H NMR (400 MHz, CDCl_3_): δ 5.46 (t, *J* = 9.8 Hz, 2H), 5.19 (t, *J* = 9.5 Hz, 1H), 5.09-5.03 (m, 2H), 5.00-4.96 (m, 1H), 4.83 (dd, *J* = 10.2, 3.7 Hz, 1H), 4.47 (d, *J* = 7.9 Hz, 1H), 4.26-4.21 (m, 2H), 4.14-4.06 (m, 2H), 4.01-3.98 (m, 1H), 3.91-3.86 (m, 0.4H), 3.52-3.40 (m, 1H), 2.07 (s, 3H), 2.04 (m, 3H), 2.019 (s, 4H), 2.01 (s, 1H), 1.99 (s, 3H), 1.73-1.64 (m, 2H), 1.50-1.47 (m, 2H), 0.91-0.86 (m, 8H); ^13^C NMR (100 MHz, CDCl_3_): δ 170.7, 170.4, 170.3, 170.2, 169.7, 169.5, 169.4, 100.9, 95.8, 73.8, 73.0, 71.8, 71.5, 71.1, 70.46, 70.40, 68.8, 68.7, 68.6, 67.3, 67.2, 62.1, 38.1, 29.8, 25.0, 24.9, 22.7, 22.5, 22.3, 20.8, 20.7, HRMS (ESI) *m/z* Calcd for C_19_H_30_O_10_, [M+NH_4_]^+^ : 436.2177, found 436.2248.

**(2*R*,3*R*,4*S*,5*R*)-2-(acetoxymethyl)-6-ethoxytetrahydro-2H-pyran-3,4,5-triyltriacetate) [Gly 7] :**The title compound was isolated by column chromatography (EtOAc/Hexane = 0.5:9.5) furnished compound **4** as sticky solid 0.05 g (55 %) as anomeric ratio (α: β = 2:1); ^1^H NMR (400 MHz, CDCl_3_): δ 5.48 (t, *J* = 9.8 Hz, 0.7 H), 5.20 (t, *J* = 9.5 Hz, 1H), 5.10-5.02 (m, 2H), 4.95 (dd, *J* = 16.9, 8.8 Hz, 1H), 4.84 (dd, *J* = 10.2, 3.7 Hz, 1H), 4.50 (d, *J* = 8.0 Hz, 1H), 4.25 (dd, *J* = 12.2, 4.5 Hz, 1H), 4.13 (dd, *J* = 12.3, 2.3 Hz, 1H), 4.08 (dd, *J* = 12.3, 2.2 Hz, 1H), 4.05-3.99 (m, 1H), 3.76-3.66 (m, 2H), 3.61–3.50 (m, 2H), 2.05 (s, 2H), 2.07 (s, 2H), 2.06 (s, 2H), 2.03 (s, 3H), 2.02 (s, 3H), 2.01 (s, 1H), 2.00 (s, 3H), 1.99 (s, 3H), 1.26 – 1.17 (m, 7H). ^13^C NMR (100 MHz, CDCl_3_) δ 170.82, 170.80 170.4, 170.3, 170.2, 169.7, 169.5, 169.4, 100.7, 95.5, 73.0, 71.9, 71.4, 71.0, 70.3, 68.8, 68.6, 67.2, 65.8, 64.2, 62.1, 62.0, 20.86, 20.85, 20.83, 20.79, 20.75, 20.73, 15.1, 15.0; HRMS (ESI) *m/z* Calcd for C_16_H_24_O_10_, [M+Na]^+^: 399.1267, found 399.1295.

(2*R*,3*R*,4*S*,5*R*)-2-(acetoxymethyl)-6-propoxytetrahydro-2H-pyran-3,4,5-triyl triacetate [Gly 8]:

The title compound was isolated by column chromatography (EtOAc/hexane = 95 : 5) furnished compound colourless sticky liquid 0.03 g in 33% yield; ^1^H NMR (400 MHz, CDCl_3_) δ 5.47 (t, *J* = 9.6 Hz, 1H), 5.19 (t, *J* = 9.6 Hz, 0.4H), 5.09-4.95 (m, 3H), 4.84 (dd, *J* = 4,10.4 Hz, 1H), 4.48 (d, *J* = 7.6 Hz, 0.4 H), 4.27-4.22 (m, 2H), 4.14-4.06 (m, 2H), 4.01 (ddd, *J* = 2.0, 4.4, 10 Hz, 1H), 3.82 (dt, *J* = 6.4, 9.6 Hz, 0.4H), 3.69-3.60 (m, 1.4H), 3.45-3.35 (m, 1.4H), 2.07-1.99 (m, 17H), 1.63-1.58 (m, 3H), 0.94-0.86 (m, 4H).^13^C NMR (100 MHz, CDCl_3_) δ 170.8, 170.7, 170.4, 170.3, 170.2, 169.7, 169.5, 169.4, 100.9, 95.7, 73.0, 71.9, 71.8, 71.5, 71.1, 70.45, 70.4, 68.8, 68.6, 67.2, 62.14, 62.10, 22.8, 22.6, 20.8, 20.78, 20.74, 20.72, 10.64, 10.60 HRMS(ESI) *m/z* Calcd for C_17_H_26_O_10_, [M+Na]+ 413.1418, found 413.1436..

(2R,3R,4S,5R)-2-(acetoxymethyl)-6-isopropoxytetrahydro-2H-pyran-3,4,5-triyltriacetate) [Gly 9]: The title compound was isolated by column chromatography (EtOAc/hexane = 1:9) furnished compound 6 as sticky solid 0.06 g in in 67 % yield; (α:β, 2:1) ^1^H NMR (400 MHz, CDCl_3_) δ 5.44 (t, *J* = 9.8 Hz, 1H), 5.19-5.15 (m, , 1H), 5.02 (dd, *J* = 20.4, 10.2 Hz, 3H), 4.91 (t, *J* = 8.8 Hz, 1H), 4.77 (dd, *J* = 10.2, 3.8 Hz, 1H), 4.52 (d, *J* = 8.0 Hz, 0.3H), 4.22 (dd, *J* = 12.3, 4.7 Hz, 2H), 4.10 – 4.03 (m, 2H), 3.90-3.80 (m, 1H), 3.66 (ddd, *J* = 9.8, 4.7, 2.3 Hz, 1H), 2.05 (s, 3H), 2.02 (s, 2H), 2.00 (s, 3H), 1.99 (s, 1H), 1.98 (s, 3H), 1.97 (s, 1H), 1.22-1.19 (m, 3H), 1.18-1.08 (m, 3H). ^13^C NMR (100 MHz, CDCl_3_) δ 170.7, 170.4, 170.3, 170.2, 169.7, 169.5, 169.3, 99.7, 94.2, 73.1, 72.9, 71.7, 71.6, 71.5, 71.0, 70.3, 68.8, 68.6, 67.2, 62.2, 62.1, 23.3, 23.1, 22.0, 21.6, 20.79, 20.7. HRMS(ESI) *m/z* Calcd for C_17_H_26_O_10_, [M+NH_4_]^+^ 408.1864, found 408.1931.

(2R,3S,4R,5R)-5-acetamido-2-(acetoxymethyl)-6-propoxytetrahydro-2H-pyran-3,4-diyl diacetate) [Gly 10]: The title compound was isolated by column chromatography (EtOAc/hexane = 1:9) furnished compound 6 as sticky solid 0.05 g in 56 % yield. ^1^H NMR (400 MHz, CDCl_3_) δ 5.68 (d, *J* = 9.4 Hz, 30H), 5.20 (t, *J* = 10.0 Hz, 29H), 5.10 (t, *J* = 9.8 Hz, 26H), 4.81 (d, *J* = 3.5 Hz, 26H), 4.32 (td, *J* = 10.2, 3.6 Hz, 28H), 4.21 (dd, *J* = 12.3, 4.6 Hz, 27H), 4.08 (dd, *J* = 12.3, 2.0 Hz, 28H), 3.95-3.91(m, 1H), 3.62 (dt, *J* = 9.5, 6.8 Hz, 1H), 3.39 (dt, *J* = 9.6, 6.6 Hz, 1H), 2.07 (s, 3H), 2.01 (d, *J* = 3.9 Hz, 6H), 1.93 (s, 3H), 1.62 (m, 2H), 0.93 (t, *J* = 7.4 Hz, 3H), ^13^C NMR (100 MHz, CDCl_3_) δ 171.1, 170.7, 169.9, 169.4, 97.2, 71.5, 70.2, 68.3, 67.8, 62.1, 52.0, 29.8, 23.2, 22.7, 20.84, 20.81, 20.7, 10.7 HRMS(ESI) *m/z* Calcd for C_17_H_27_NO_9_, [M+H]^+^ 390.1759, found 390.1828.

(2R,3S,4R,5R,6S)-5-acetamido-2-(acetoxymethyl)-6-(isopentyloxy)tetrahydro-2H-pyran-3,4-diyl diacetate [Gly 11]: The title compound was isolated by column chromatography (EtOAc/hexane = 0.5 : 9.5) furnished compound colourless sticky liquid 0.03 g in 42 % yield; ^1^H NMR (400 MHz, CDCl_3_) δ 5.63 (d, *J* = 9.5 Hz, 1H), 5.20 (t, *J* = 9.6 Hz, 1H), 5.10 (t, *J* = 9.7 Hz, 1H), 4.82 (d, *J* = 3.6 Hz, 1H), 4.33 (td, *J* = 10.1, 3.6 Hz, 1H), 4.23 (dd, *J* = 12.3, 4.7 Hz, 1H), 4.10 (dd, *J* = 12.3, 2.1 Hz, 1H), 3.95-3.92 (m, 1H), 3.73 (dt, *J* = 9.8, 7.0 Hz, 1H), 3.48-3.42 (m, 2H), 2.09 (s, 3H), 1.53-1.48 (m, 2H), 0.89-0.87 (m, 1H) 2.03-2.02 (m, 5H), 1.95 (s, 2H), 0.93 (d, *J* = 6.6 Hz, 5H).^13^C NMR (100 MHz, CDCl_3_) 171.5, 171.1, 170.8, 169.9, 97.3, 71.6, 68.3, 67.9, 67.0, 62.2, 52.0, 38.2, 25.2, 23.3, 22.7, 22.5, 20.8, 20.7, HRMS(ESI) *m/z* Calcd for C_19_H_31_NO_9_, [M+H]+ 418.2072, found 418.2095.

(2R,3S,4R,5R,6S)-5-acetamido-2-(acetoxymethyl)-6-butoxytetrahydro-2H-pyran-3,4-diyl diacetate [Gly 12]: The title compound was isolated by column chromatography (EtOAc/Hexane = 1: 9) furnished compound 6 as sticky solid 0.1 g in in 57 % yield.; ^1^H NMR (400 MHz, CDCl_3_) δ 5.68 (d, *J* = 9.5 Hz, 1H), 5.18 (t, *J* = 10.0 Hz, 1H), 5.08 (t, *J* = 9.8 Hz, 1H), 4.80 (d, *J* = 3.6 Hz, 1H), 4.31 (td, *J* = 10.2, 3.7 Hz, 1H), 4.21 (dd, *J* = 12.3, 4.6 Hz, 1H), 4.07 (dd, *J* = 12.3, 2.1 Hz, 1H), 3.92 (ddd, *J* = 9.9, 4.4, 2.2 Hz, 1H), 3.67 (dt, *J* = 9.7, 6.7 Hz, 1H), 3.42 (dt, *J* = 9.8, 6.5 Hz, 1H), 2.07 (s, 3H), 2.01 (s, 3H),1.99 (s, 3H), 1.93 (s, 3H), 1.61-1.54 (m, 2H), 1.39 (dd, *J* = 7.3, 3.3 Hz, 1H), 1.35 (dd, *J* = 7.4, 3.7 Hz, 1H), 0.93 (t, *J* = 7.4 Hz, 3H). ^13^C NMR (100 MHz, CDCl_3_) δ 171.4, 170.7, 169.9, 169.4, 97.2, 71.5, 68.3, 67.8, 62.1, 52.0, 31.4, 23.2, 20.83, 20.80, 20.7, 19.4, 13.8. HRMS(ESI) *m/z* Calcd for C_18_H_29_NO_9_, [M+H]+ 404.1915, found 404.2033.

**Characterization of glycoside derivatives by quadrupole/Q-TOF mass spectrometer with an ESI source determined using HRMS spectra.**


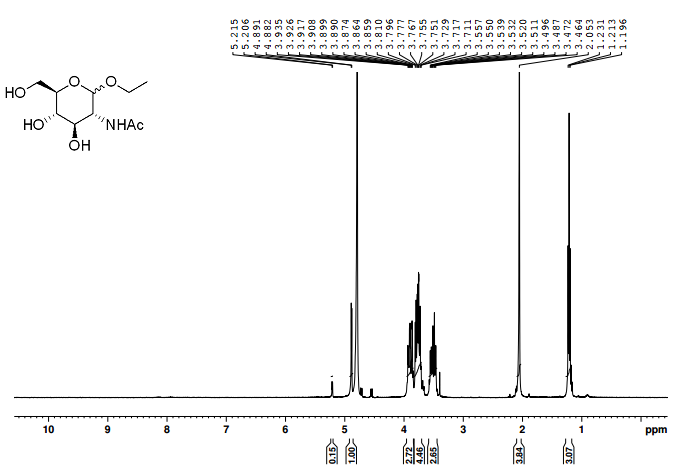


**Gly 1**


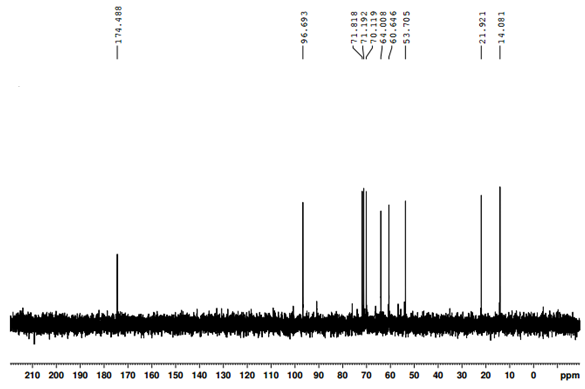


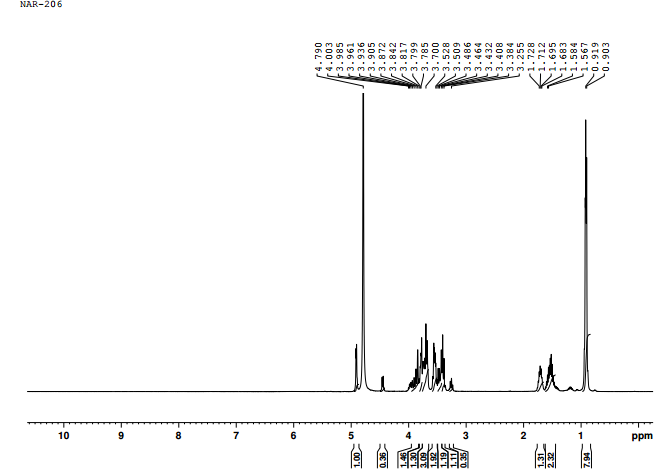

**Gly 2**


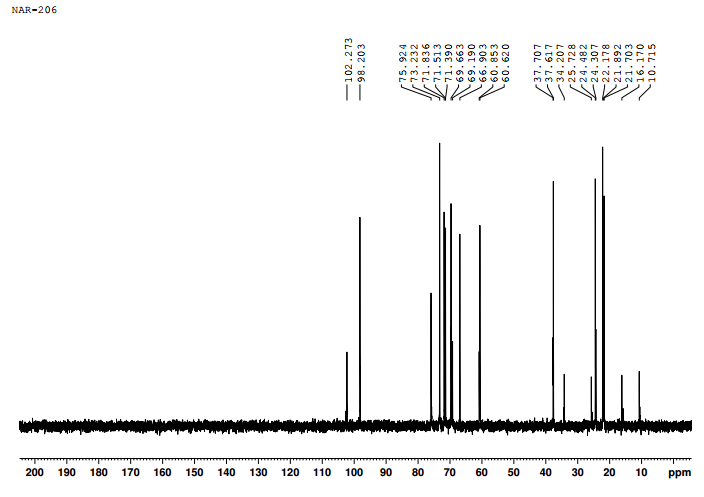


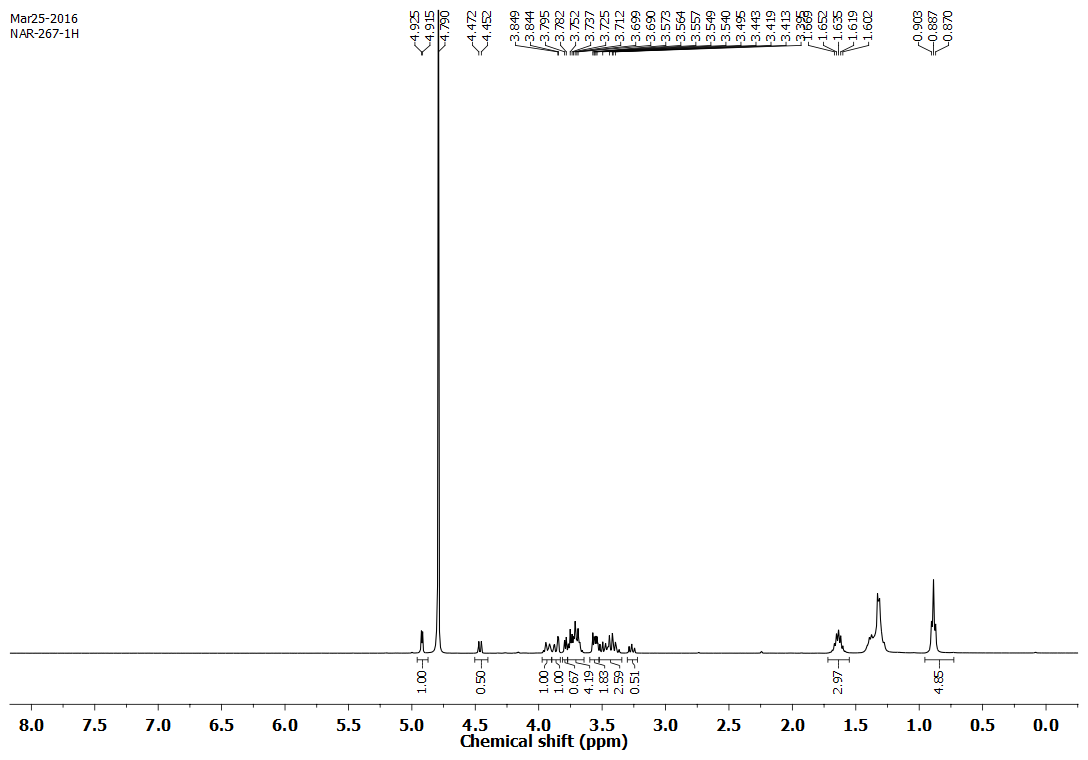

**Gly 3**


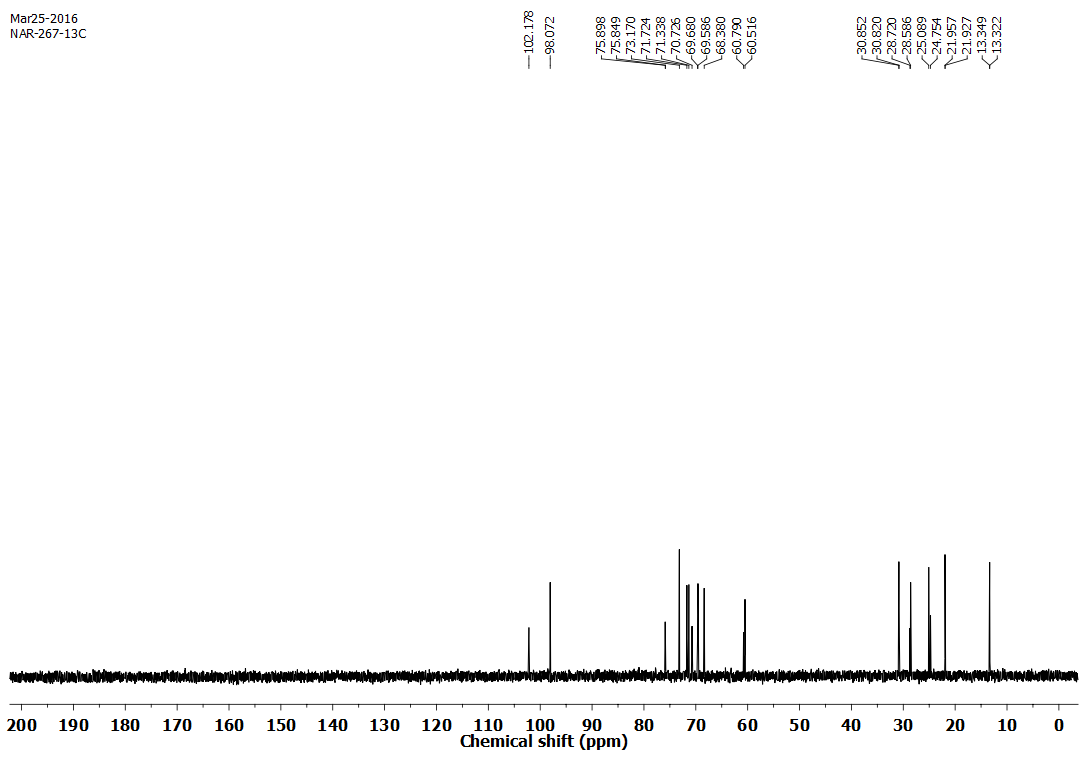


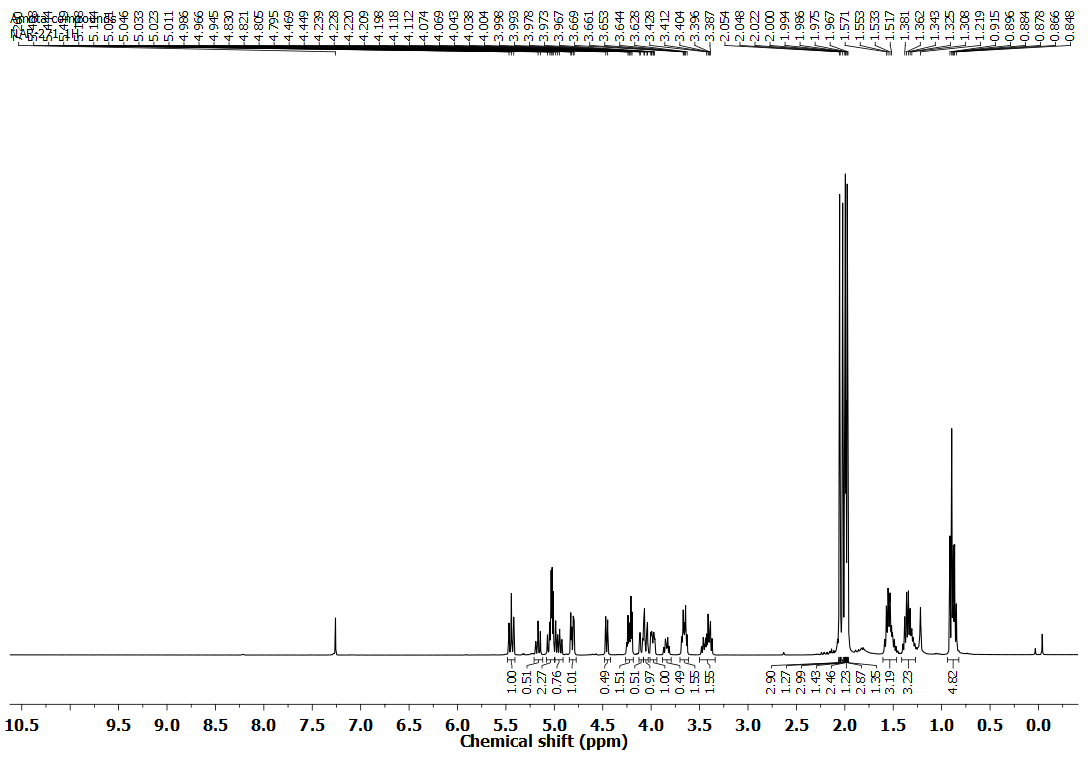

**Gly 4**


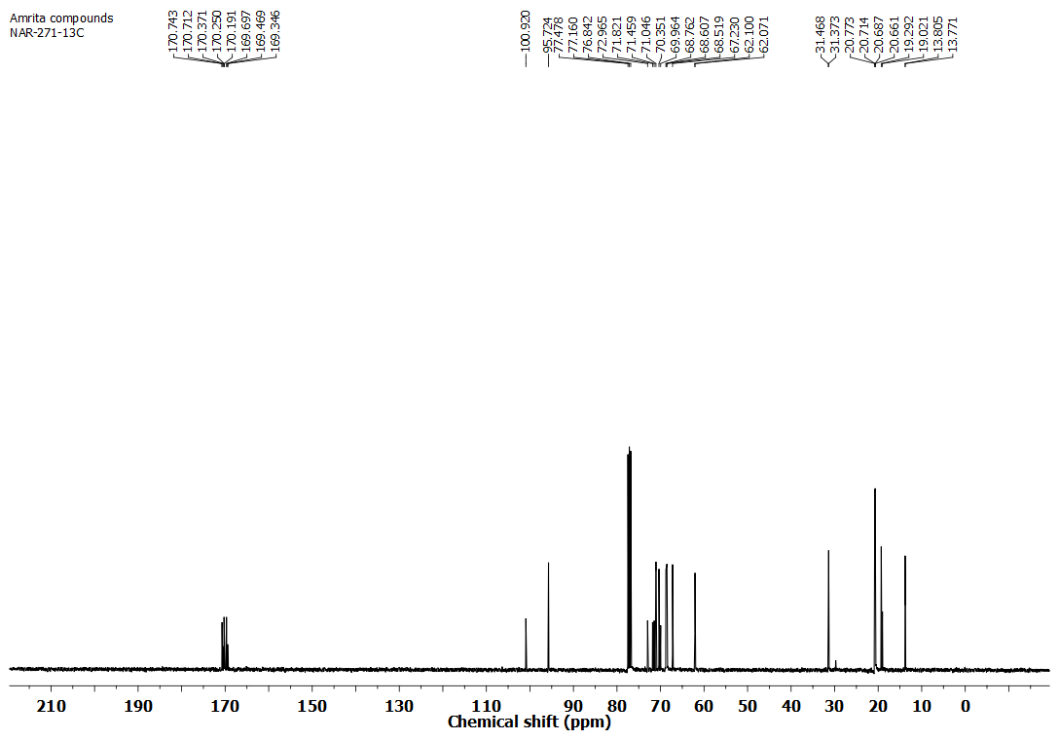


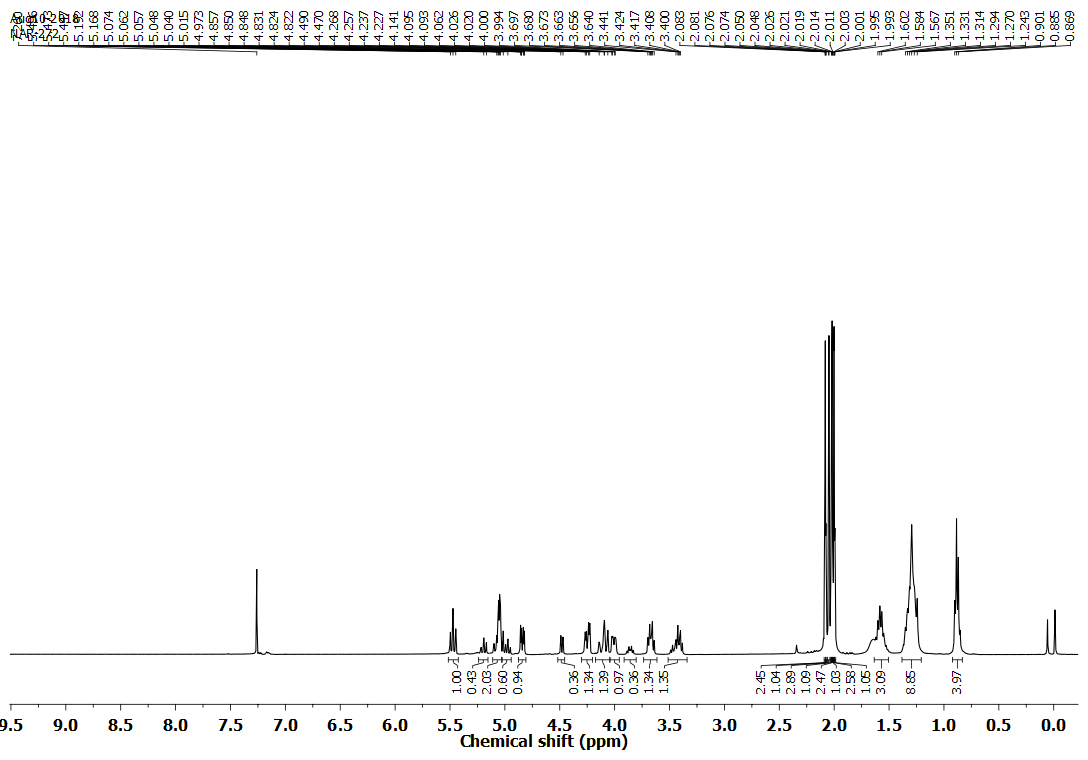

**Gly 5**


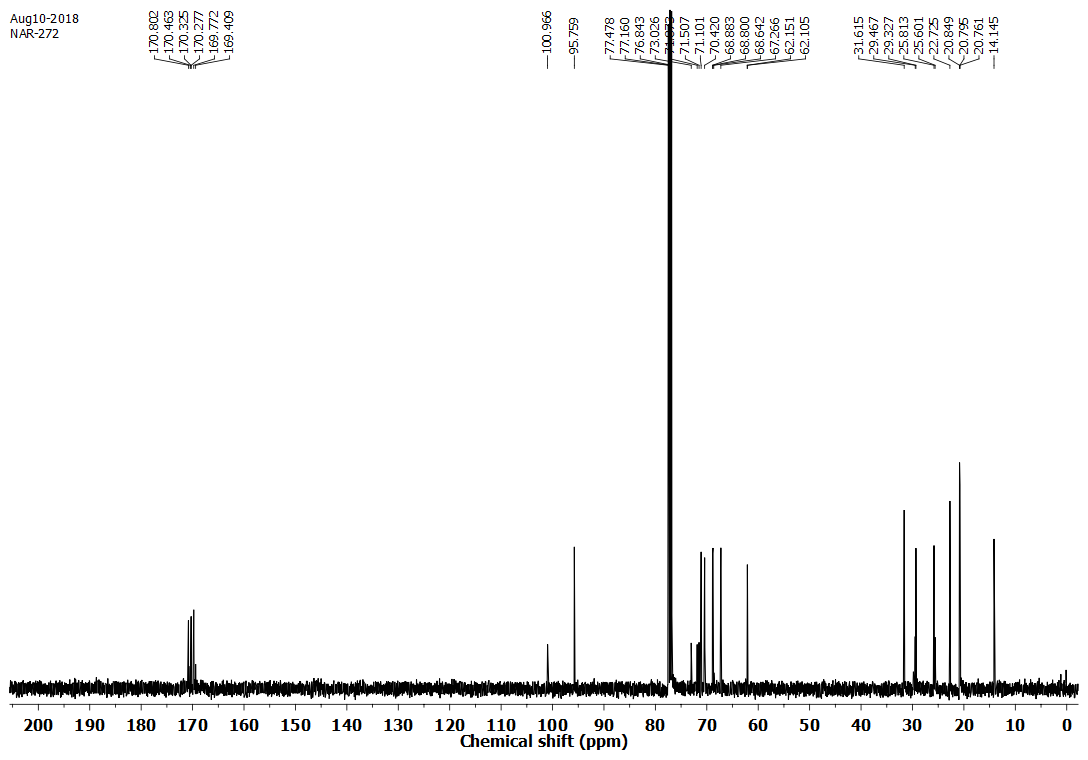


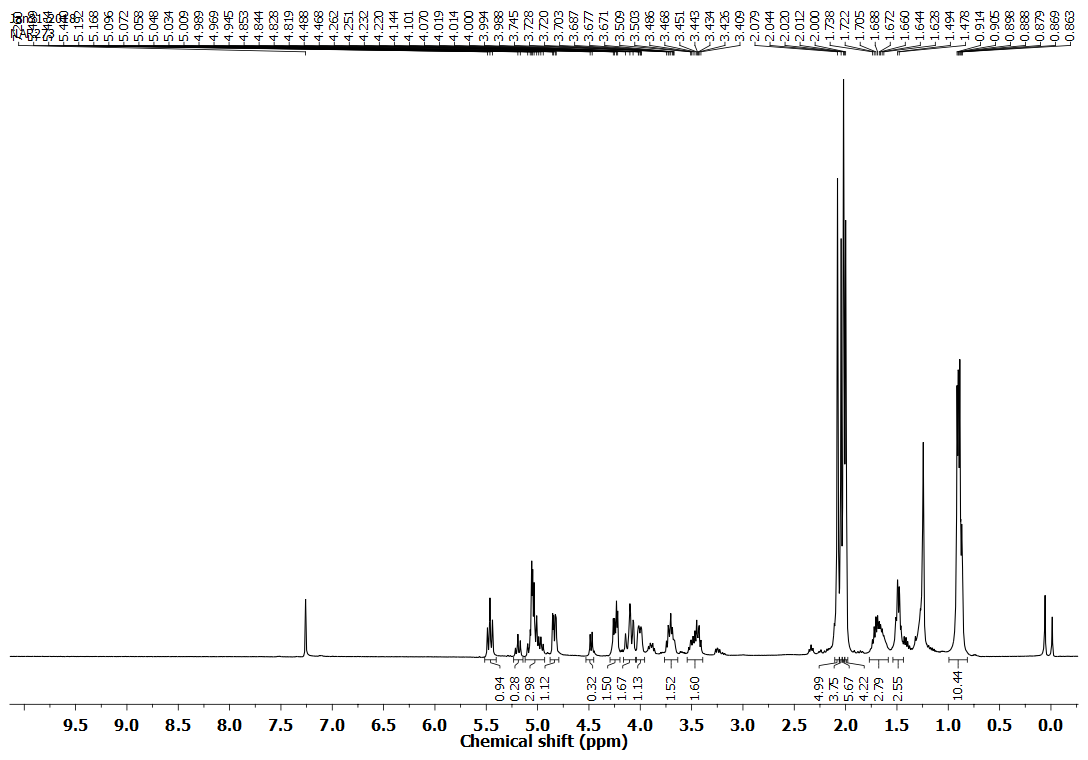

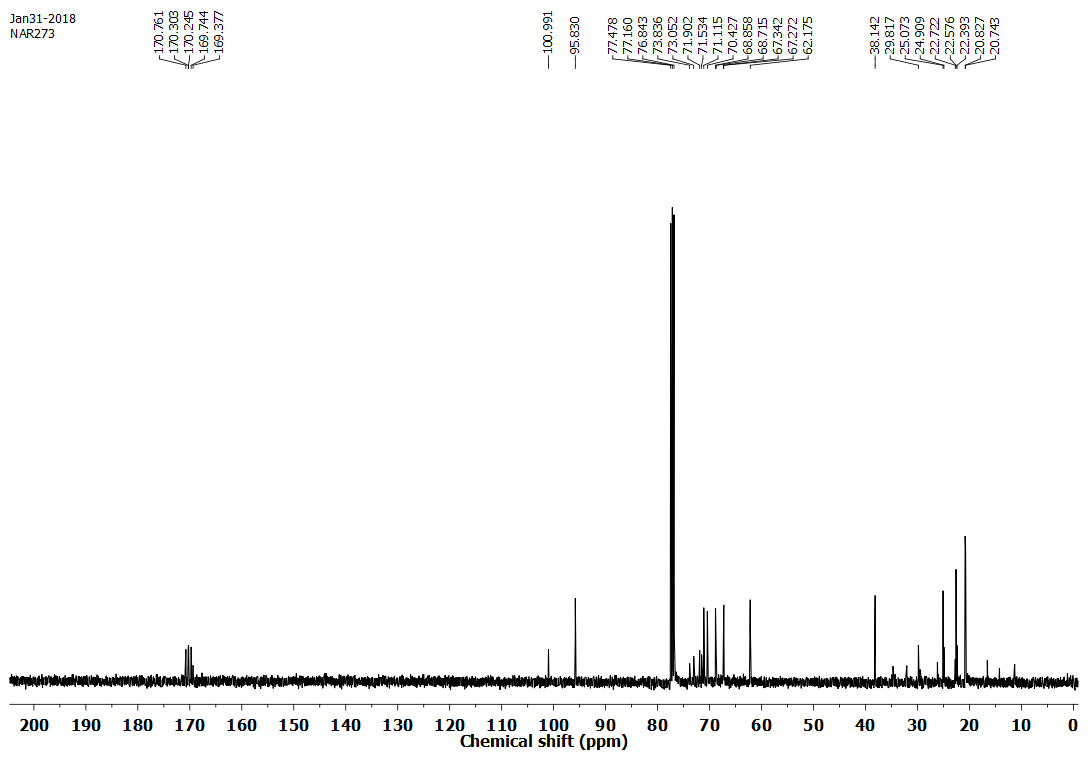

**Gly 6**


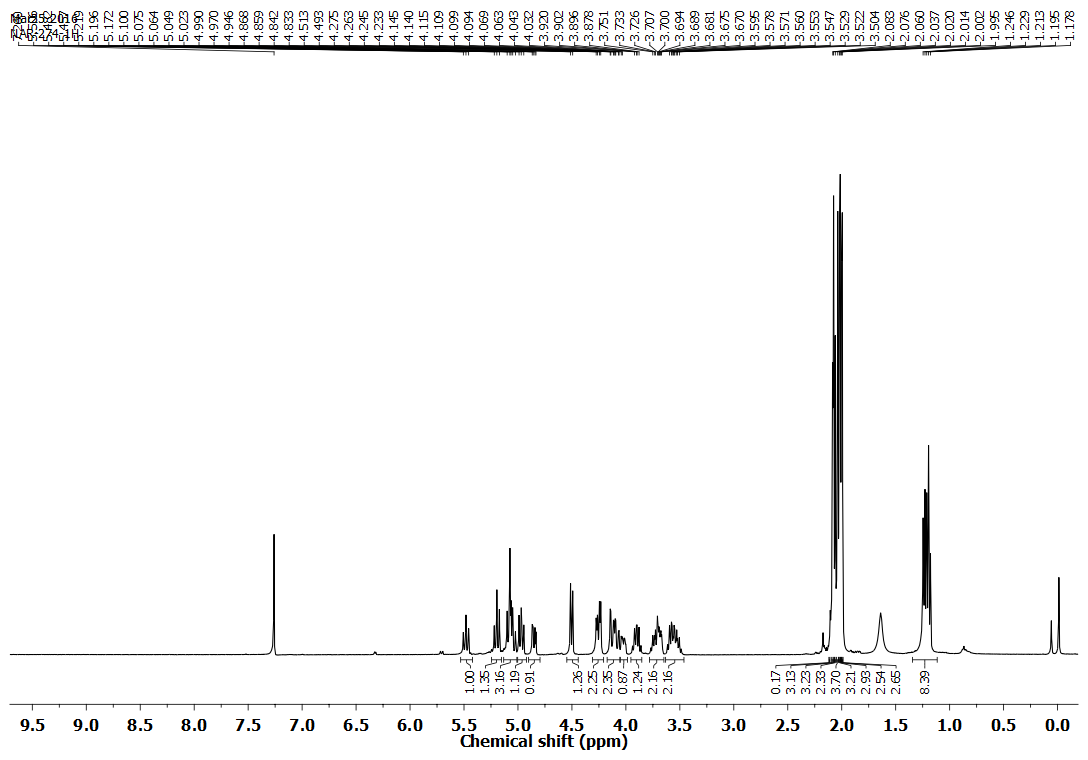

**Gly 7**


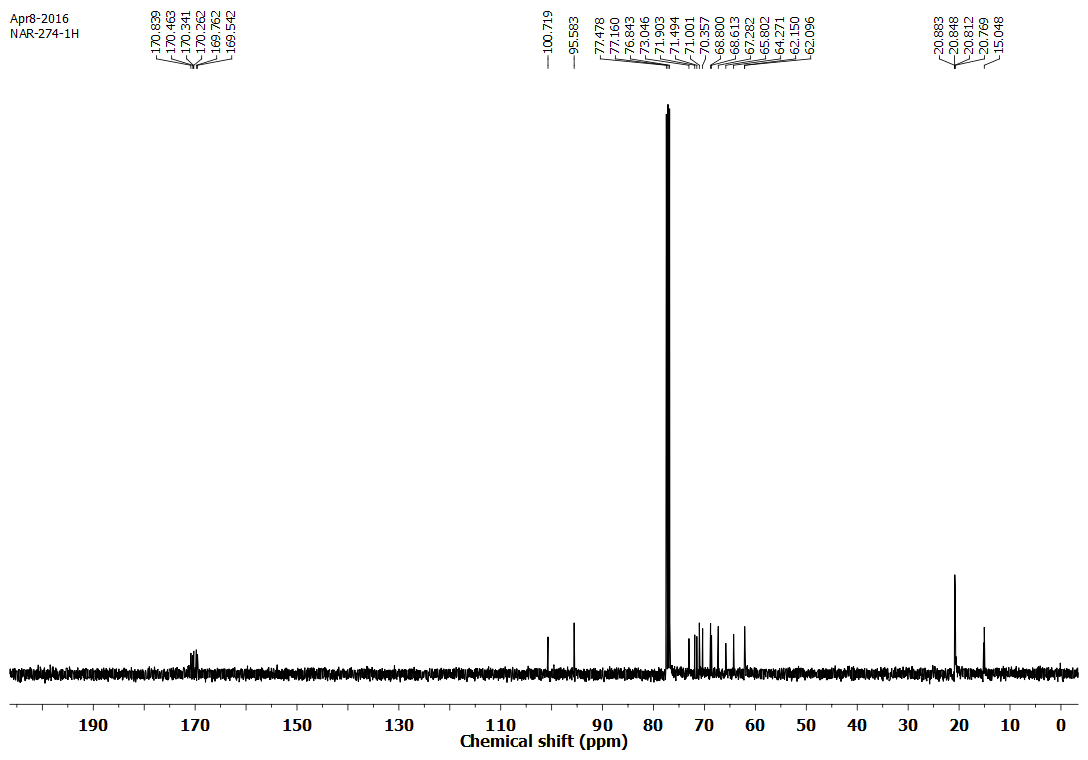


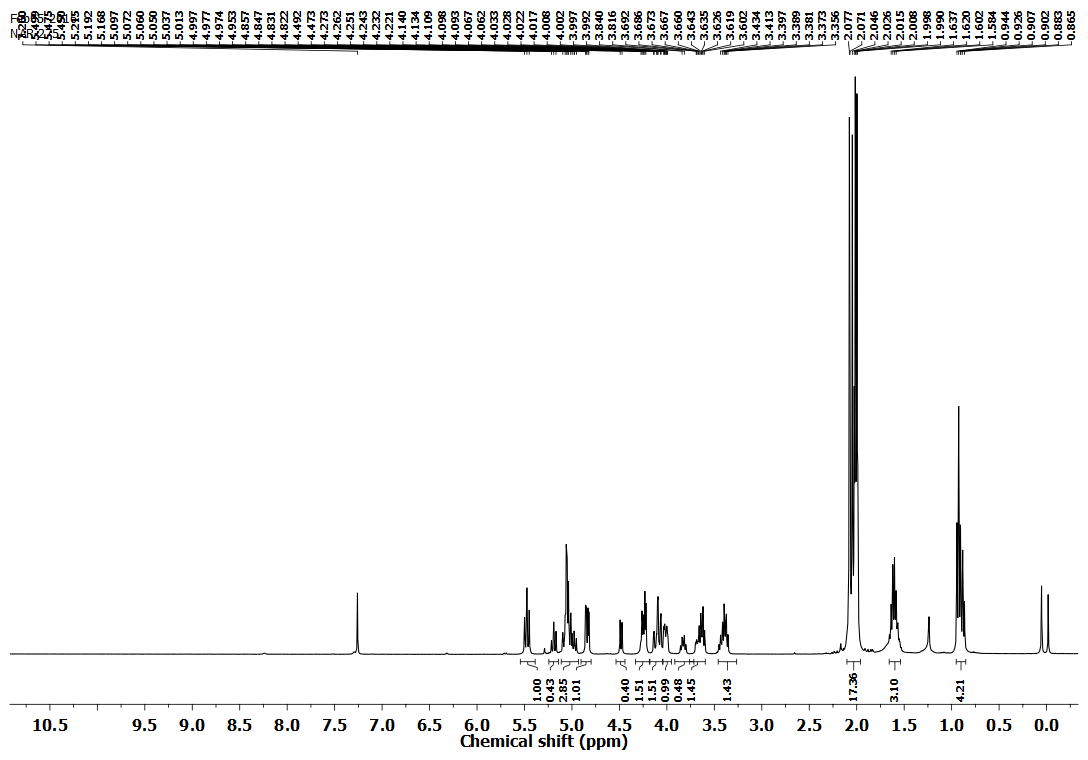

**Gly 8**


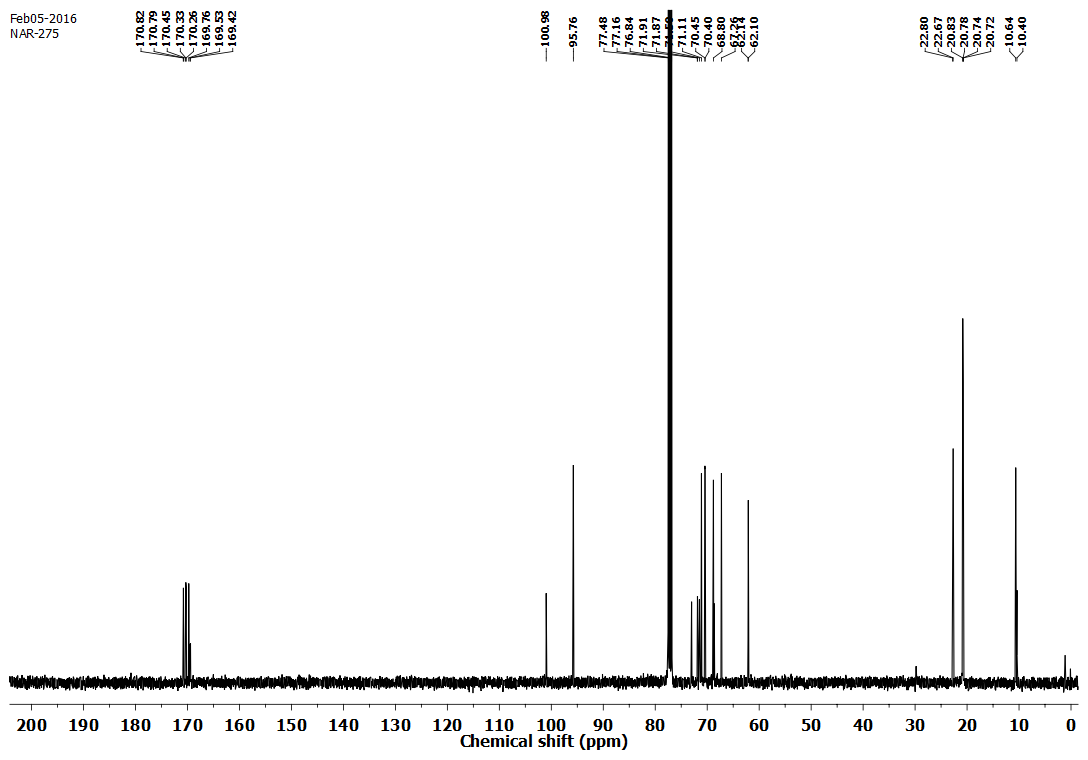


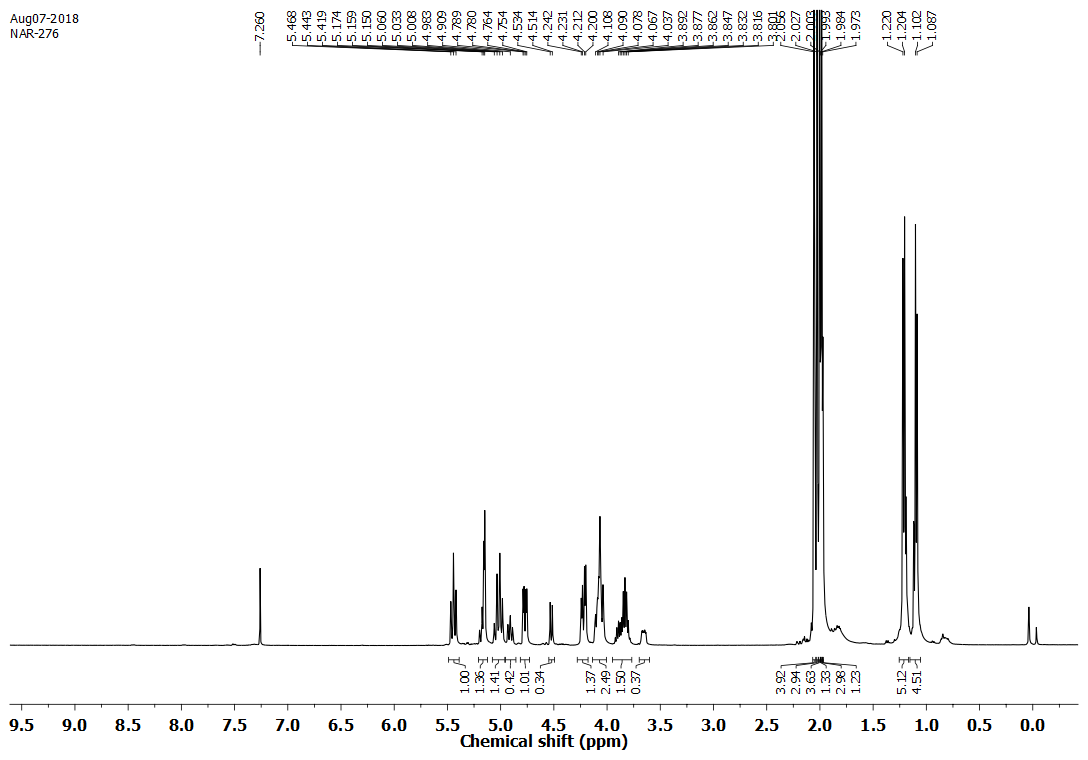

**Gly 9**


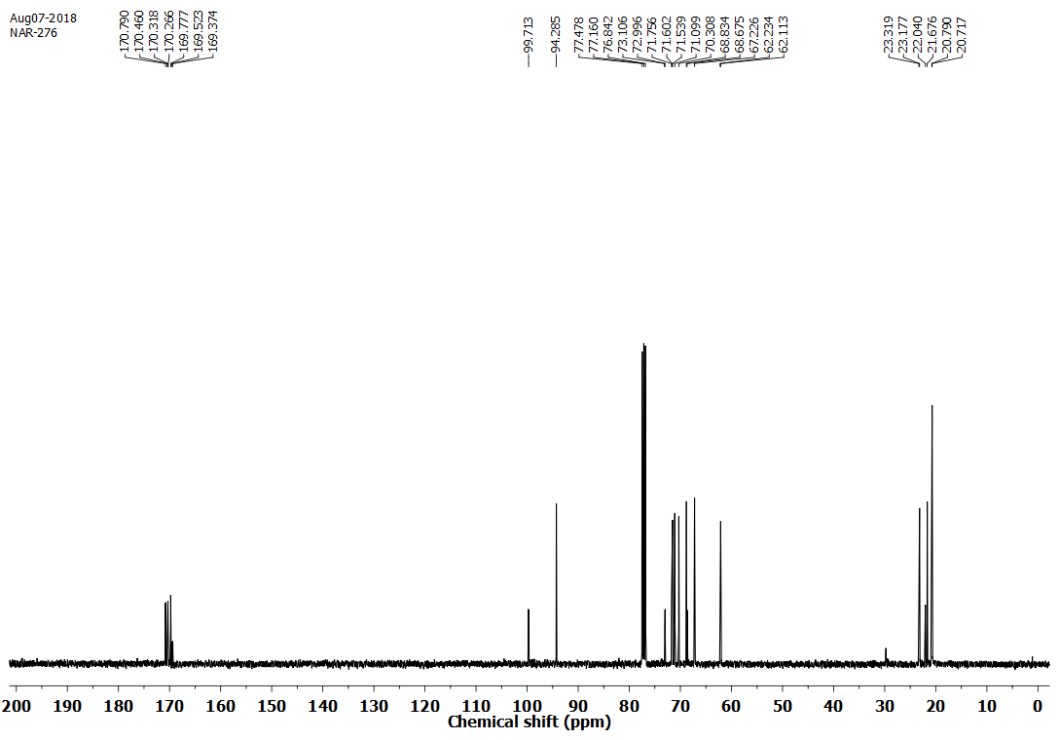


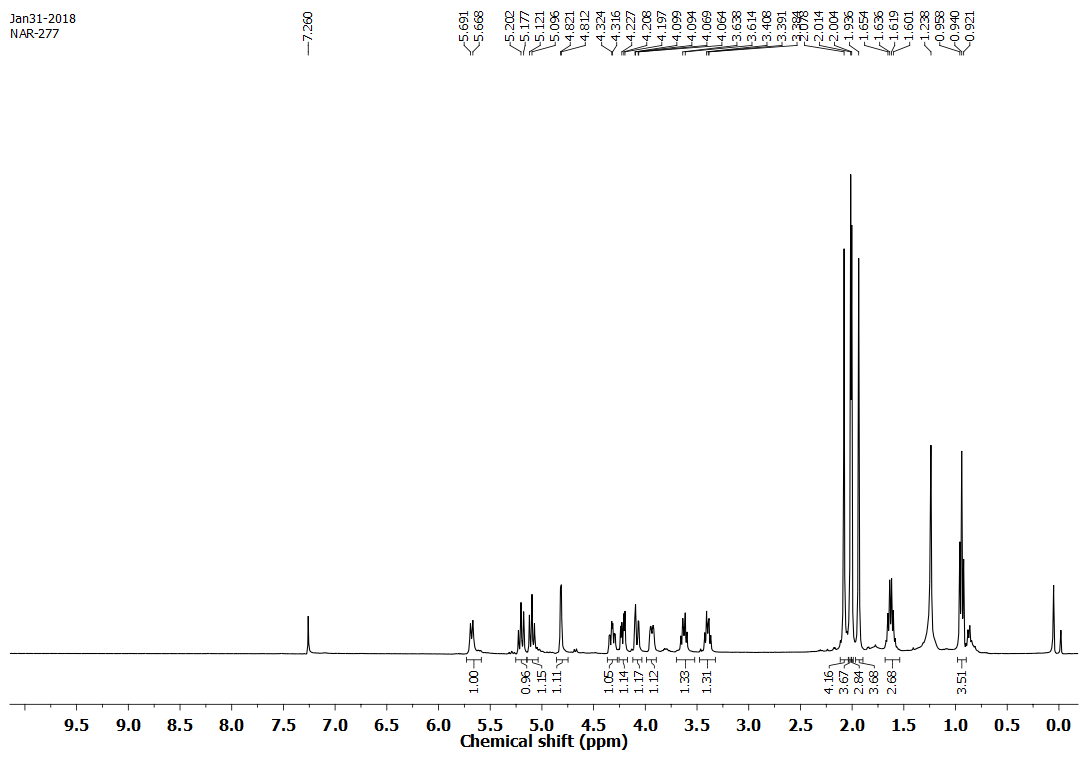

**Gly 10**


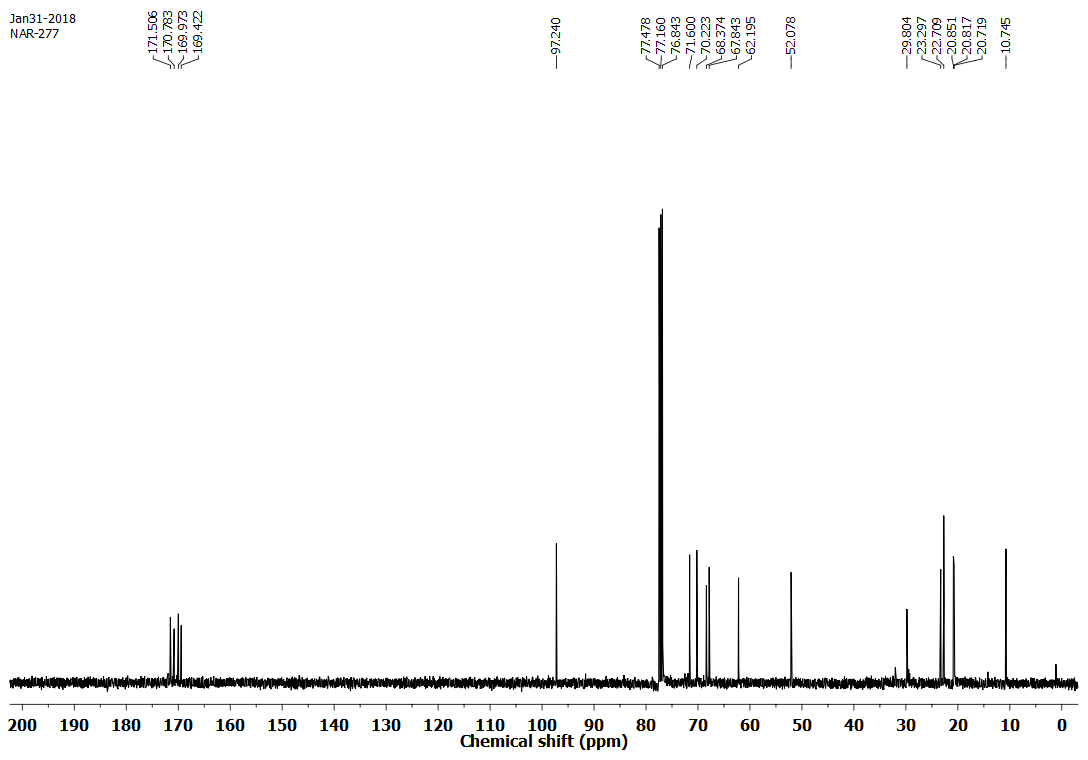


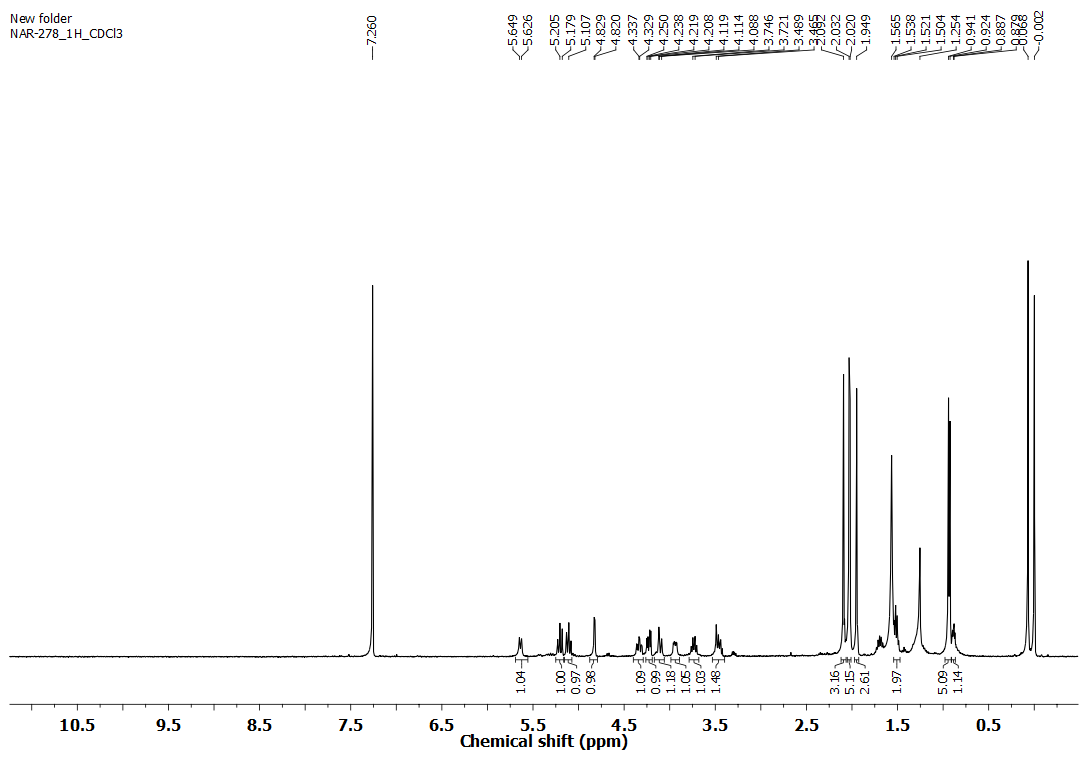

**Gly 11**


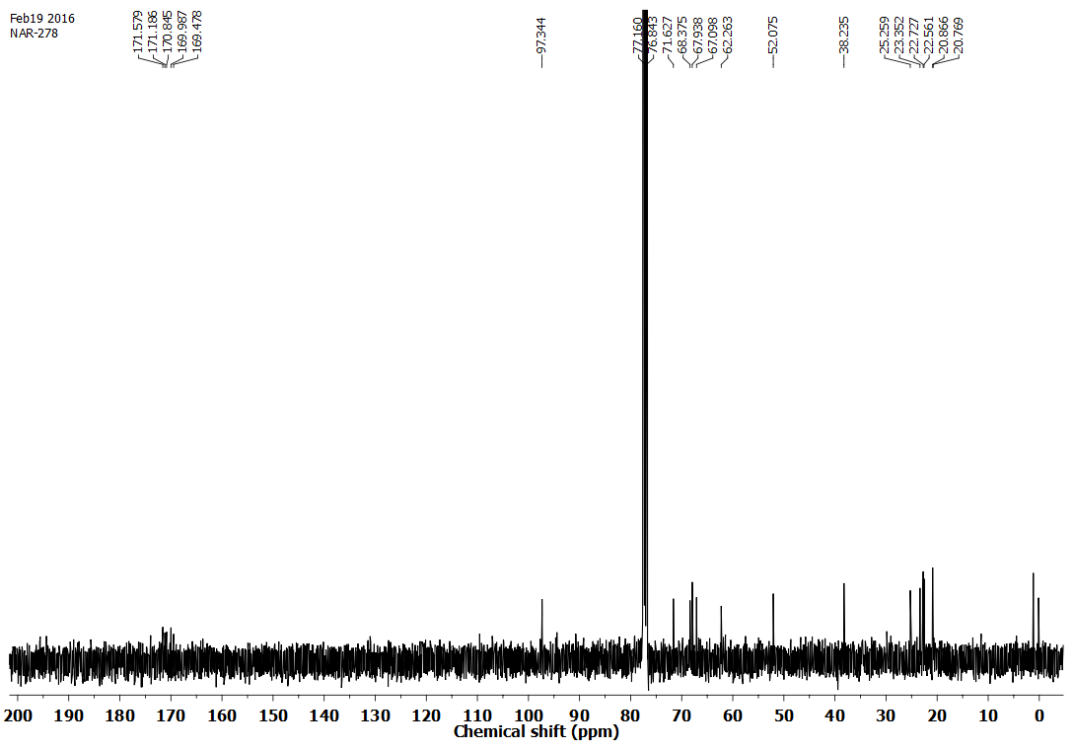


***
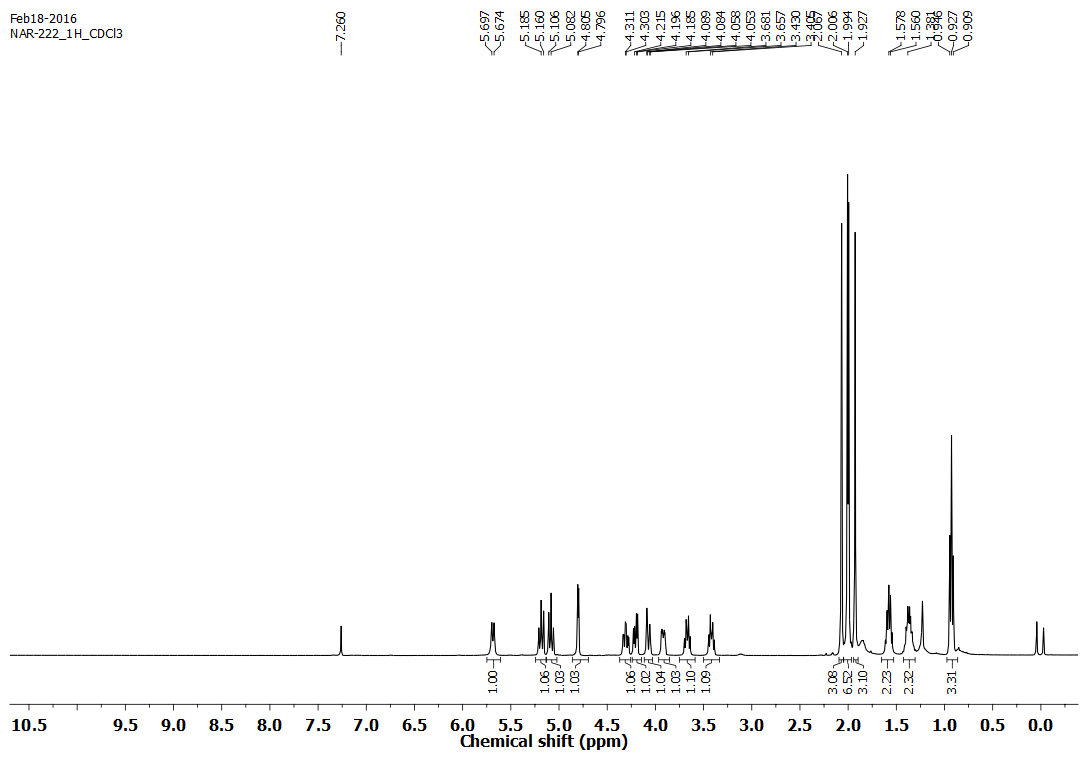
***

**Gly 12**


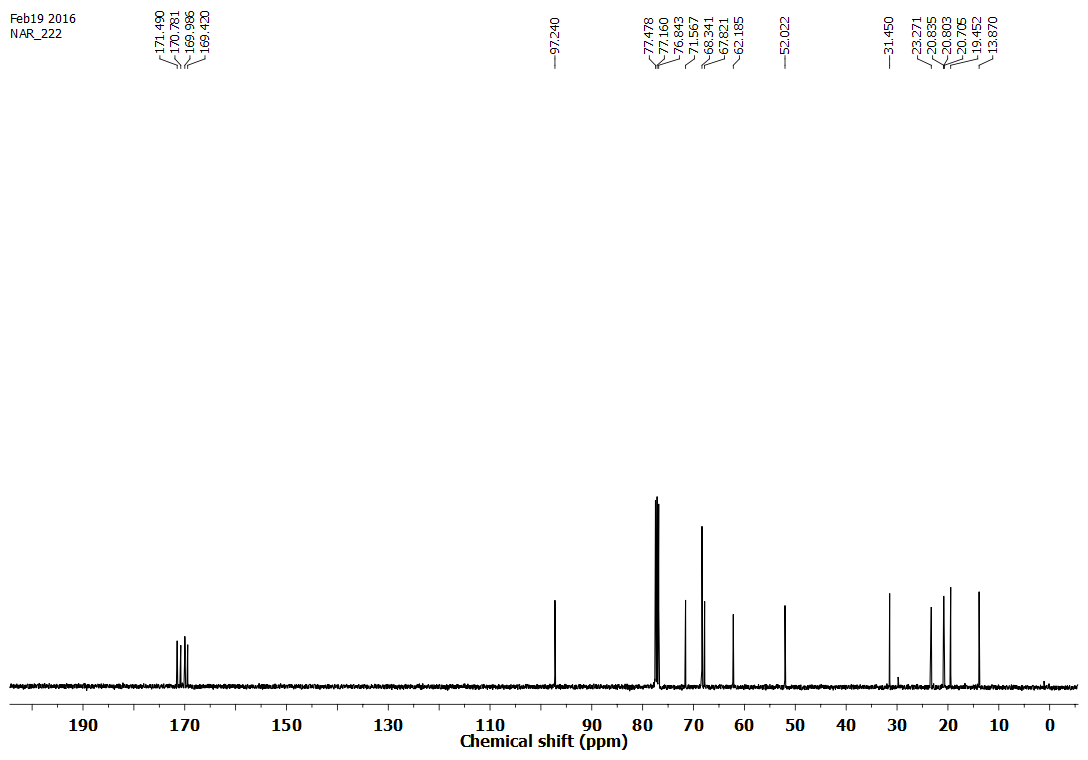

Supplement: Supplementary file 1 [file DataSheet_1.docx]
